# Defining the ligand-dependent proximatome of the sigma 1 receptor

**DOI:** 10.3389/fcell.2023.1045759

**Published:** 2023-06-07

**Authors:** Jing Zhao, Rajalakshmi Veeranan-Karmegam, Frederick C. Baker, Barbara A. Mysona, Pritha Bagchi, Yutao Liu, Sylvia B. Smith, Graydon B. Gonsalvez, Kathryn E. Bollinger

**Affiliations:** ^1^ Department of Ophthalmology, Medical College of Georgia at Augusta University, Augusta, GA, United States; ^2^ Culver Vision Discovery Institute, Augusta, GA, United States; ^3^ Department of Cellular Biology and Anatomy, Medical College of Georgia at Augusta University, Augusta, GA, United States; ^4^ Emory Integrated Proteomics Core, Emory University, Atlanta, GA, United States

**Keywords:** molecular chapterone, ERGIC, cholesterol, PCSK9, LDLR, secretome

## Abstract

Sigma 1 Receptor (S1R) is a therapeutic target for a wide spectrum of pathological conditions ranging from neurodegenerative diseases to cancer and COVID-19. S1R is ubiquitously expressed throughout the visceral organs, nervous, immune and cardiovascular systems. It is proposed to function as a ligand-dependent molecular chaperone that modulates multiple intracellular signaling pathways. The purpose of this study was to define the S1R proximatome under native conditions and upon binding to well-characterized ligands. This was accomplished by fusing the biotin ligase, Apex2, to the C terminus of S1R. Cells stably expressing S1R-Apex or a GFP-Apex control were used to map proximal proteins. Biotinylated proteins were labeled under native conditions and in a ligand dependent manner, then purified and identified using quantitative mass spectrometry. Under native conditions, S1R biotinylates over 200 novel proteins, many of which localize within the endomembrane system (endoplasmic reticulum, Golgi, secretory vesicles) and function within the secretory pathway. Under conditions of cellular exposure to either S1R agonist or antagonist, results show enrichment of proteins integral to secretion, extracellular matrix formation, and cholesterol biosynthesis. Notably, Proprotein Convertase Subtilisin/Kexin Type 9 (PCSK9) displays increased binding to S1R under conditions of treatment with Haloperidol, a well-known S1R antagonist; whereas Low density lipoprotein receptor (LDLR) binds more efficiently to S1R upon treatment with (+)-Pentazocine ((+)-PTZ), a classical S1R agonist. Furthermore, we demonstrate that the ligand bound state of S1R correlates with specific changes to the cellular secretome. Our results are consistent with the postulated role of S1R as an intracellular chaperone and further suggest important and novel functionalities related to secretion and cholesterol metabolism.

## Introduction

The sigma 1 receptor (S1R) is a small (25kD), ubiquitously expressed, transmembrane protein that is localized within the endoplasmic reticulum and its mitochondria-associated membranes (Hayashi and Su, 2005; Hayashi and Su, 2007). Studies also show that it can translocate to nuclear and plasma membranes under certain conditions (Su et al., 2010; Mavlyutov et al., 2015a; Tsai et al., 2015). S1R has been the subject of intense pharmacologic analysis over the past several decades due to the therapeutic potential of its ligands. In many instances, S1R-mediated therapeutics are advancing from bench to bedside. For example, clinical trials targeting S1R for treatment of neurodegenerative diseases including Amyotrophic Lateral Sclerosis (ALS) and Huntingtons Disease are ongoing (Arnold, 2021). In addition, promising preclinical studies indicate that S1R may offer a treatment target for visual disorders including glaucoma, retinal degeneration and traumatic optic neuropathy (Mavlyutov et al., 2015b; Nguyen et al., 2015; Wang et al., 2016; Sambo et al., 2017; Geva et al., 2021; Li et al., 2021). Furthermore, in recent studies, S1R was shown to link the SARS-CoV2 replicase/transcriptase complex to the ER membrane by binding directly to nonstructural protein 6 (NSP 6) (Gordon et al., 2020). Therefore, S1R ligands may provide antiviral activity against severe acute respiratory syndrome CoV-2 (SARS-CoV-2) (Gordon et al., 2020; Vela, 2020). Finally, there is interest in using S1R ligands for treating and imaging cancer (Happy et al., 2015; Das et al., 2016).

Despite intense interest, the molecular mechanisms that underlie effects of S1R ligands are not well understood. In general, agonists for S1R show pro-survival effects whereas antagonists inhibit tumor cell proliferation and induce apoptosis (Maurice and Su, 2009). Studies indicate that S1R functions as a “pluripotent modulator” of multiple signaling pathways and therefore affects a wide range of cellular activities including calcium homeostasis, ion channel regulation, and responses to ER and oxidative stress (Hayashi and Su, 2003). The proposed general mechanism for S1R function is through protein-protein interactions (Hayashi and Su, 2007). In support of this paradigm, S1R has been reported to bind to at least 49 different proteins that generally show diverse structure and function (Schmidt and Kruse, 2019). However, published experiments used to support direct interactions between S1R and other proteins have been mainly limited to low-throughput, candidate-based methods. These include co-immunoprecipitation and resonance energy transfer experiments (Navarro et al., 2010; Balasuriya et al., 2013; Tsai et al., 2015). Previous studies have also utilized proximity ligation assays, but few have combined these evaluations with high-throughput proteomic analyses.

In this study, a cell line-based proximity biotin labeling assay was developed and combined with proteomic identification (Rhee et al., 2013). The promiscuous biotin ligase Apex2 fused to the C-terminus of S1R was used to label proximal proteins under native conditions and in a ligand dependent manner (Lam et al., 2015; Hung et al., 2016). The biotinylated proteins were then purified and identified using quantitative mass spectrometry. The labeling radius of Apex2 is thought to be around 20 nm (Trinkle-Mulcahy, 2019). Thus, although this strategy does not necessarily identify direct protein interaction partners, biotinylated proteins are proximal to the bait protein and may be present in a complex along with it. We refer to the proteins identified in this manner as the S1R-Apex proximatome. Under native conditions, we find that S1R-Apex proximatome contains over 200 novel proteins, many of which localize within the endomembrane system (ER, Golgi, secretory vesicles) and function within the cellular secretory apparatus. In addition, under conditions of cellular exposure to either S1R agonist or antagonist we identify proximatome changes that highlight the molecular pathways critical to S1R-mediated ligand-dependent effects. These include proteins involved in cholesterol and lipid metabolism as well as matricellular and extracellular proteins. Thus our results offer insight into the cellular changes that accompany S1R ligand binding.

## Results

### Defining the proximatome of the Sigma1 receptor

**FIGURE 1:**
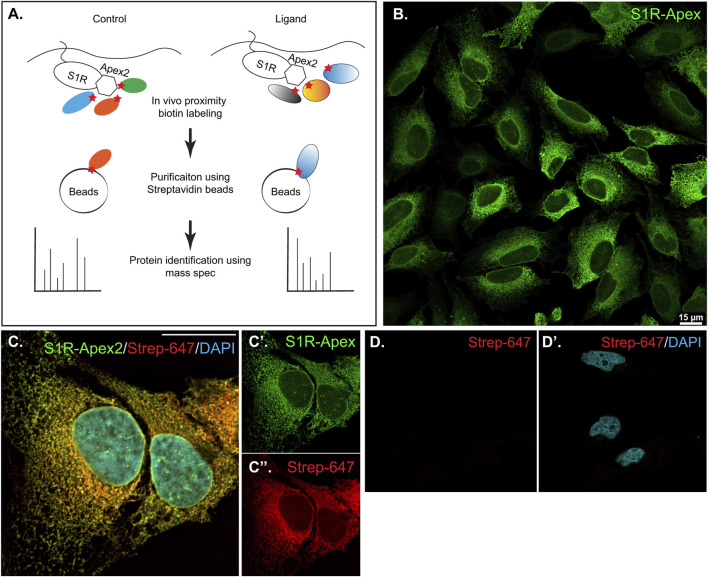
Generation and validation of S1R-Apex cells. **(A)** Schematic of the proximity biotinylation strategy **(B)** HeLa cells stably expressing S1R-Apex were fixed and processed for immunofluorescence using a V5 antibody (green). S1R-Apex localizes around the nuclear envelope and to ER tubules. **(C)** HeLa cells stably expressing S1R-Apex were fixed and processed for immunofluorescence using a V5 antibody (green). The cells were also incubated with Streptavidin-647 (red) to reveal the localization of biotinylated proteins and were counterstained using DAPI (cyan). Biotinylated proteins co-localize with S1R-Apex. **(D)** Control HeLa cells not expressing a biotin ligase were processed using Streptavidin-647 (red). The cells were counterstained with DAPI (cyan). Minimal Streptavidin signal is observed in these cells. The scale bar in B is 15 microns and the scale bar in C is 20 microns.

In order to identify proteins central the ligand-dependent function of Sigma1 receptor (S1R), we employed a proximity biotinylation approach. With this strategy, the protein of interest is tagged with a promiscuous biotin ligase. Proteins that are present within 20 nm of the tagged protein become biotinylated *in vivo* and can be easily purified using streptavidin conjugated beads (Figure 1A) (Cho et al., 2020). One of the main advantages of this approach is that because the biotin-streptavidin interaction is extremely strong, the purification can be done using harsh wash buffers. This minimizes binding of non-specific proteins to beads, thus reducing the potential for false-positive hits (Samavarchi-Tehrani et al., 2020). For our studies, we chose to tag S1R at the C-terminus with Apex2 (Schmidt et al., 2016; Mavlyutov et al., 2017; Schmidt et al., 2018). We chose this approach because previous studies have shown that S1R-Apex is functional and produces the expected localization pattern (Mavlyutov et al., 2017). In addition, a structural analysis of S1R revealed that only a few N-terminal residues are exposed within the cytoplasm. The bulk of the protein, including the C-teminus, resides within the ER (Schmidt et al., 2016). Thus, tagging S1R on the C-terminus with Apex2 was the most logical approach. A V5 epitope was incorporated into the tag in order to enable Western blotting and immunofluorescence analysis using commercially available antibodies. A construct expressing GFP-Apex was used as the negative control.

HeLa cells stably expressing S1R-Apex and GFP-Apex were generated using CRISPR-based incorporation of the constructs at the *AAVS1* safe harbor locus using a published protocol (Oceguera-Yanez et al., 2016). Immunostaining of cells expressing S1R-Apex produced the expected localization pattern (Figure 1B). In order to validate the proximity biotinylation approach, cells expressing S1R-Apex were labeled using biotin phenol. Control cells that did not express a biotin ligase were similarly treated. The cells were then processed for immunofluorescence using a V5 antibody for detecting the Apex tagged protein, and Streptavidin conjugated Alexa647 for detecting biotinylated proteins. In contrast to control cells, robust biotinylation signal was observed in cells expressing S1R-Apex (Figures 1C, D). Furthermore, the biotinylation signal co-localized with the V5 signal for S1R-Apex (Figure 1C). The biotinylation pattern is consistent with the published localization of S1R to ER and nuclear membranes (Jiang et al., 2006; Mavlyutov et al., 2015a; Mavlyutov et al., 2017). By contrast, cells expressing GFP-Apex displayed a nuclear and cytoplasmic biotinylation pattern (Supplementary Figure S1). Based on these results, we conclude that S1R-Apex is localizing as expected and biotinylating proximal proteins.

**FIGURE 2:**
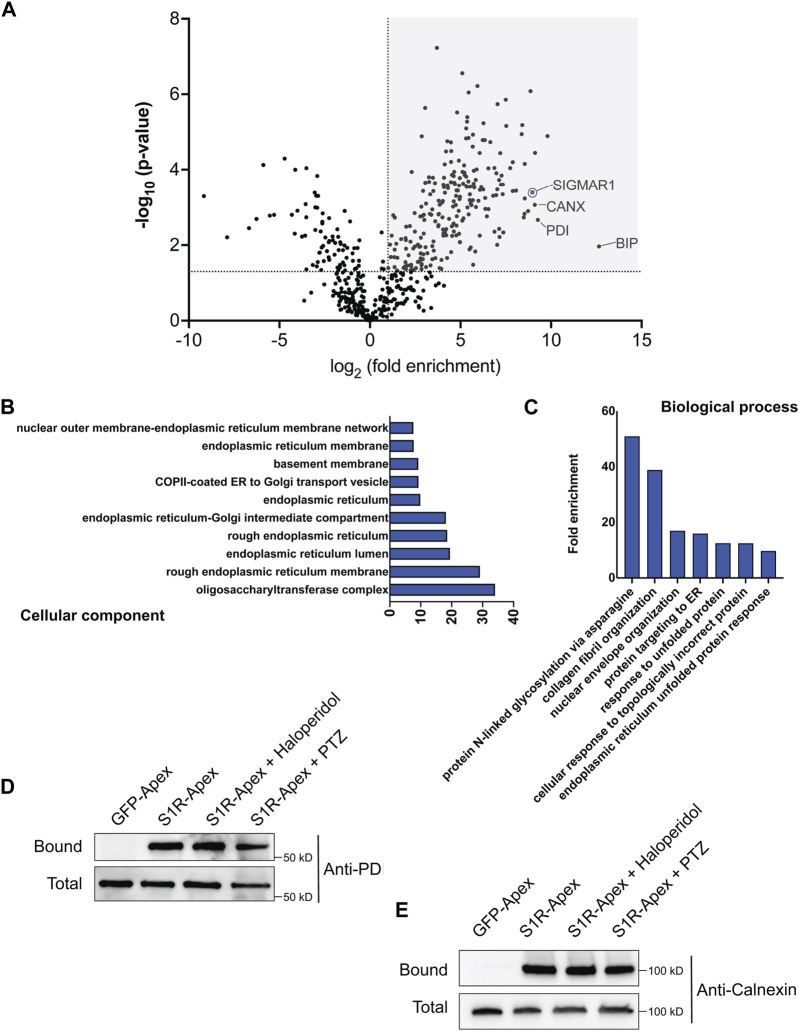
The S1R proximatome. **(A)** A volcano plot depicting the S1R proximatome. A line demarcating 2-fold enrichment and a *p*-value of 0.05 is shown. The grey shaded box indicates proteins that are at least 2-fold enriched with S1R in comparison to the GFP-Apex control with a *p*-value of at least 0.05. **(B)** A Cellular Component GO analysis of S1R proximal proteins. **(C)** A Biological Process GO analysis of S1R proximal proteins. **(D,E)** Biotinylated proteins were purified from HEK293T cells stably expressing either GFP-Apex or S1R-Apex using streptavidin conjugated beads. Biotinylated proteins were also purified from HEK293T cells stably expressing S1R-Apex that were treated with either Haloperidol or (+)-PTZ. The bound proteins were eluted and analyzed by Western blotting using an antibody against PDI **(D)** or Calnexin **(E)**. S1R-Apex expressed in HEK293T cells is able to biotinylate PDI and Calnexin. This proximal association is not altered by treatment with Haloperidol or (+)-PTZ.

Next, cells expressing GFP-Apex and S1R-Apex were grown and labeled for proteomics analysis. Lysates were prepared from the labeled cells and the biotinylated proteins were purified using high-capacity Streptavidin agarose beads. After extensive wash steps, the bound proteins were eluted using Trypsin digestion and processed for mass-spectrometry. The entire experiment was done in triplicate. Proteins that were enriched at least two-fold in the S1R-Apex pellet in comparison to GFP-Apex and had a *p*-value of at least 0.05 were considered to be part of the S1R proximatome (Figure 2A; Supplementary Table S1). Using these criteria, the S1R proximatome contains 233 specific proteins in HeLa cells. The top 60 candidates are shown in Table 1.

**TABLE 1:** A list of the top 60 S1R proximal proteins.

Protein name	Log_2_ fold enrichment	Protein name	Log_2_ fold enrichment
78 kDa glucose-regulated protein	12.645	Dolichyl-diphosphooligosaccharide--protein glycosyltransferase subunit 1	6.984
Protein disulfide-isomerase	9.272	Dolichyl-diphosphooligosaccharide--protein glycosyltransferase subunit STT3B	6.973
Urotensin-2	9.149	Endoplasmic reticulum resident protein 44	6.941
Aspartyl/asparaginyl beta-hydroxylase	9.081	Thioredoxin domain-containing protein 5	6.932
Protein disulfide-isomerase A6	8.929	Follistatin-related protein 1	6.855
Calreticulin	8.909	Peptidyl-prolyl cis-trans isomerase B	6.774
Sigma non-opioid intracellular receptor 1	8.794	LDLR chaperone MESD	6.711
Calnexin	8.595	Coiled-coil domain-containing protein 47	6.691
Endoplasmin	8.538	Endoplasmic reticulum-Golgi intermediate compartment protein 1	6.592
Glucosidase 2 subunit beta	8.470	Transmembrane emp24 domain-containing protein 9	6.565
Protein disulfide-isomerase A4	8.235	Mesencephalic astrocyte-derived neurotrophic factor	6.519
Protein disulfide-isomerase A3	8.223	Peptidyl-prolyl cis-trans isomerase FKBP10	6.493
Neutral alpha-glucosidase AB	8.071	Transmembrane protein 109	6.413
Ubiquitin carboxyl-terminal hydrolase 32; Ubiquitin carboxyl-terminal hydrolase 6	7.900	Integrin beta-1	6.345
Cytoskeleton-associated protein 4	7.886	Very-long-chain enoyl-CoA reductase	6.340
Reticulocalbin-1	7.806	Malectin	6.326
Procollagen-lysine,2-oxoglutarate 5-dioxygenase 2	7.777	Dolichyl-diphosphooligosaccharide--protein glycosyltransferase subunit 2	6.312
Hypoxia upregulated protein 1	7.746	Transmembrane emp24 domain-containing protein 10	6.276
Inactive phospholipase C-like protein 2	7.702	Serpin H1	6.259
Calumenin	7.601	Insulin-like growth factor-binding protein 7	6.231
Transmembrane protein 43	7.562	Prolyl 4-hydroxylase subunit alpha-1	6.213
Endoplasmic reticulum resident protein 29	7.506	Erlin-1	6.178
UDP-glucose:glycoprotein glucosyltransferase 1	7.371	Inhibitor of nuclear factor kappa-B kinase-interacting protein	6.151
Myeloid-derived growth factor	7.339	Peroxidasin homolog	6.126
Dolichyl-diphosphooligosaccharide--protein glycosyltransferase 48 kDa subunit	7.295	Integrin alpha-11	6.092
Homeobox protein Hox-A9	7.102	Protein jagunal homolog 1	6.053
Peptidyl-prolyl cis-trans isomerase FKBP9	7.062	Translocon-associated protein subunit alpha	6.050
Surfeit locus protein 4	7.024	Signal peptidase complex subunit 3	5.987
Protein ERGIC-53	7.004	Beta-2-microglobulin; Beta-2-microglobulin form pI 5.3	5.964
Dolichyl-diphosphooligosaccharide--protein glycosyltransferase subunit 1	6.984	Transmembrane protein 33	5.883

S1R is known to exist as a trimeric complex *in vivo* (Schmidt et al., 2016). Thus, peptides corresponding to S1R should be highly enriched in the S1R-Apex dataset. This was indeed the case (Figure 2A). In addition, the ER chaperone BiP (GRP78), a known S1R interacting partner, was also specifically enriched in the S1R-Apex pellet (Figure 2A) (Hayashi and Su, 2007). Previous studies have shown that S1R localizes to the endoplasmic reticulum (ER), to nuclear lamellae, to sites of ER-mitochondria contact and to the plasma membrane (Hayashi and Su, 2007; Su et al., 2010; Tsai et al., 2015; Mavlyutov et al., 2017). Consistent with these studies, a “cellular component” Gene Ontology (GO) analysis indicates that the S1R proximatome is highly enriched for proteins that localize to the nuclear membrane, to the ER lumen and membrane, and to organelles involved in protein secretion. (Figure 2B). Previous studies have implicated a role for S1R in the ER stress response and as a potential molecular chaperone. In line with these findings, a “biological process” GO analysis is enriched for terms such as “protein targeting to ER” and “response to unfolded protein.” In addition, proteins with a role in N-linked glycosylation are highly enriched within the S1R proximatome (Figure 2C).

In order to validate these results, we generated HEK293T cells that stably express either GFP-Apex or S1R-Apex integrated at the *AAVS1* safe harbor locus (Oceguera-Yanez et al., 2016). Two ER proteins that were highly enriched in the S1R proximatome were Protein Disulfide Isomerase (PDI) and the chaperone Calnexin. Consistent with the proteomics results, PDI and Calnexin were specifically biotinylated and precipitated in HEK293T cells expressing S1R-Apex (Figures 2D, E). Biotinylation of these proteins by S1R-Apex was unchanged upon treating cells with either Haloperidol, an S1R antagonist, or with (+)-PTZ, an S1R agonist (Figures 2D, E).

We next determined whether PDI and Calnexin were capable of co-precipitating with S1R. Using a standard co-immunoprecipitation protocol involving a lysis buffer containing 0.2% CHAPS, we were able to detect a small amount of co-precipitaitng Calnexin but not PDI (Supplementary Figure S1B and data not shown). Surprisingly, using this approach, we were not able to efficiently co-precipitate Bip along with S1R-Apex (Supplementary Figure S1B). Thus, although Calnexin, PDI and Bip were biotinylated by S1R-Apex, detecting these interactions via co-immunoprecipitation proved much more challenging. It is possible that the interactions are transient or weak and are thus not retained during the process of co-immunoprecipitation.

### S1R-apex biotinylates components of the ER translocation machinery

**FIGURE 3:**
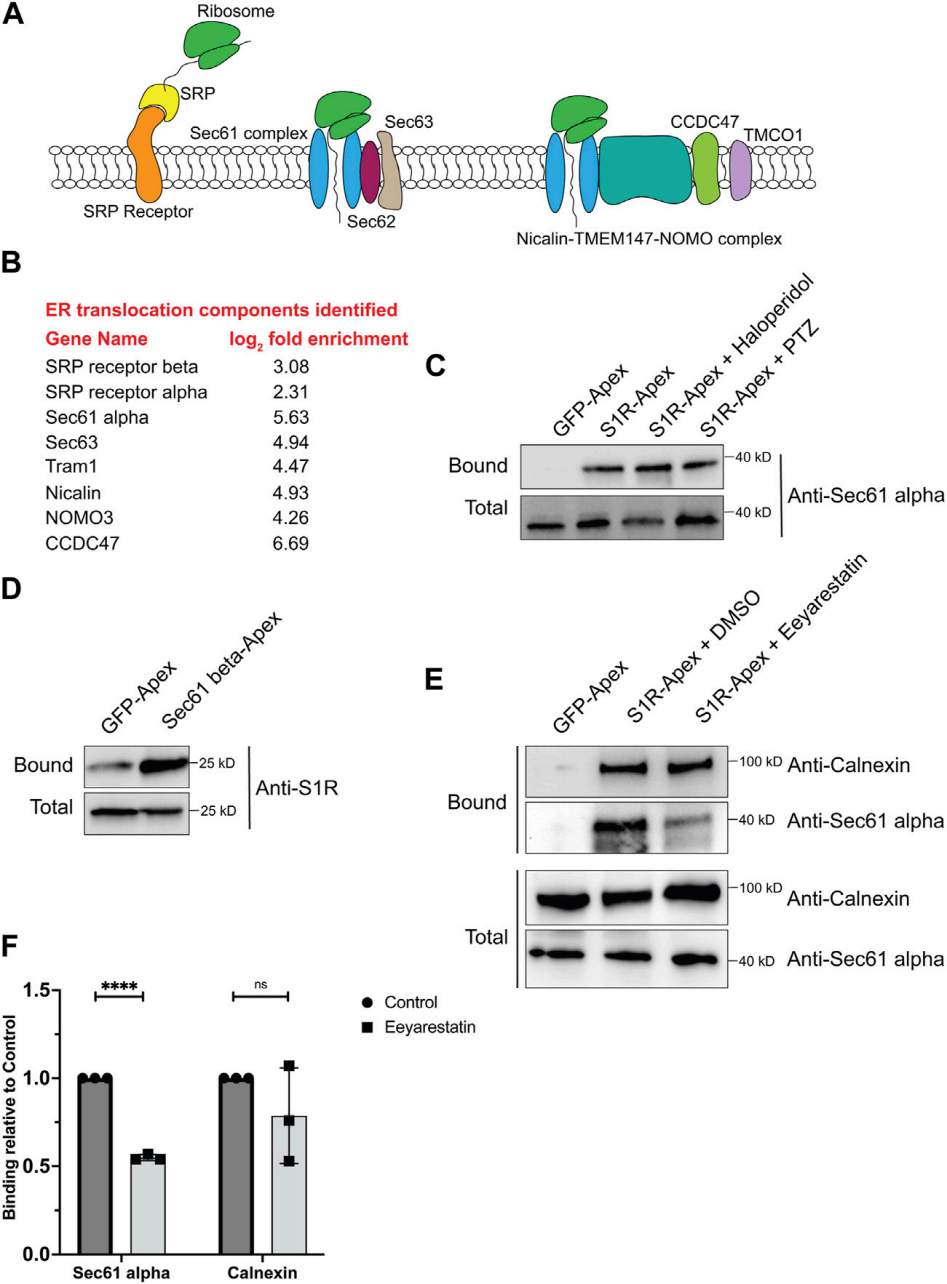
S1R biotinylates components of the ER translocation complex. **(A)** A schematic of the components involved in translocation of proteins into the ER. **(B)** A list of ER translocation components identified in the S1R proximatome. **(C)** Biotinylated proteins were purified from HEK293T cells stably expressing either GFP-Apex or S1R-Apex that were untreated or treated with either Haloperidol or (+)-PTZ. The proteins were purified using streptavidin conjugated beads. The bound proteins were eluted and analyzed by Western blotting using an antibody against Sec61alpha. S1R-Apex expressed in HEK293T cells biotinylates Sec61alpha. **(D)** HEK293T cells were transiently transfected with a construct expressing either GFP-Apex or Sec61 beta-Apex. Biotinylated proteins were purified using streptavidin conjugated beads, the bound proteins were eluted and analyzed by Western blotting using an antibody against endogenous S1R. Endogenous S1R is biotinylated by Sec61 beta-Apex, a component of the ER translocation complex. **(E)** HEK293T cells stably expressing GFP or S1R-Apex were either untreated (DMSO) or treated with the drug Eeyarestatin. Eeyarestatin blocks the transport of proteins across the Sec61 translocation channel. Biotinylated proteins were purified using streptavidin conjugated beads and the bound proteins were eluted and analyzed by Western blotting using antibodies against Sec61 alpha and Calnexin. **(F)** The above experiment was repeated in triplicate and the binding of S1R-Apex with Sec61 alpha and Calnexin was quantified. Unpaired *t* tests were used for these analyses; ****p < 0.0001, ns = not significant. Blocking protein transport across the Sec61 channel disrupts the proximal association between S1R and Sec61 alpha.

For proteins that contain a signal sequence, import into the ER occurs co-translationally. Once the signal sequence has been translated, it is bound by the signal recognition particle (SRP). This complex is then docked onto the surface of the ER by binding to the SRP receptor. Next, the docked ribosome associates with the Sec61 translocation complex and its accessory proteins. This enables the translating peptide to be imported into the ER. Once in the ER, luminal chaperones such as BiP and Calnexin help fold the protein into its native conformation (Figure 3A) (Lang et al., 2017; Linxweiler et al., 2017). Multi-pass transmembrane proteins are imported into the ER with the aid of the Nicalin-TMEM147-NOMO complex (McGilvray et al., 2020). Numerous proteins within the S1R proximatome function at various steps in the ER translocation process (Figure 3B). This finding was quite intriguing given the proposed function of S1R as a molecular chaperone. These results position S1R at the entry way into the ER, an ideal location for a molecular chaperone.

In order to validate these findings, we focused our studies on Sec61alpha, the protein that forms the central translocation channel. Consistent with the proteomics result, Sec61alpha was specifically detected in the S1R-Apex pellet from HEK293T cells (Figure 3C). As with PDI and Calnexin, the biotinylation of Sec61alpha by S1R-Apex was unchanged upon treating cells with either Haloperidol or (+)-PTZ. As further validation of this result, we determined whether endogenous untagged S1R was also present in a complex with the ER translocation channel. For this experiment, HEK293T cells were transiently transfected with either GFP-Apex or Sec61beta-Apex. Sec61beta-Apex was chosen for this experiment because this construct has been used in a previous publication (Lee et al., 2016). In addition, we were concerned that tagging Sec61alpha with Apex might compromise its function and would negatively impact import of proteins into the ER. After binding and wash steps, the bound proteins were eluted and analyzed by Western blotting using an antibody against S1R. Consistent with our proteomics results which suggests proximity between S1R-Apex and the ER translocation channel, significantly more S1R was biotinylated by Sec61beta-Apex in comparison to GFP-Apex (Figure 3D).

We next determined whether the proximal association between S1R and the Sec61 complex was sensitive to active protein import into the ER. The drug Eeyarestatin is known to inhibit Sec61 alpha, and as a consequence, ER protein import is blocked (Wang et al., 2008; Wang et al., 2010; Gamayun et al., 2019). Interestingly treating cells with Eeyarestatin reduced the biotinylation of Sec61alpha by S1R-Apex (Figures 3E, F). The biotinylation of Calnexin by S1R-Apex was also somewhat reduced. However, these results were not statistically significant (Figures 3E, F). Based on these results, we conclude that S1R localizes promixal to the ER translocation machinery and likely does so in an import-dependent manner.

### S1R-apex biotinylates components at the ER-Golgi intermediate compartment

**FIGURE 4:**
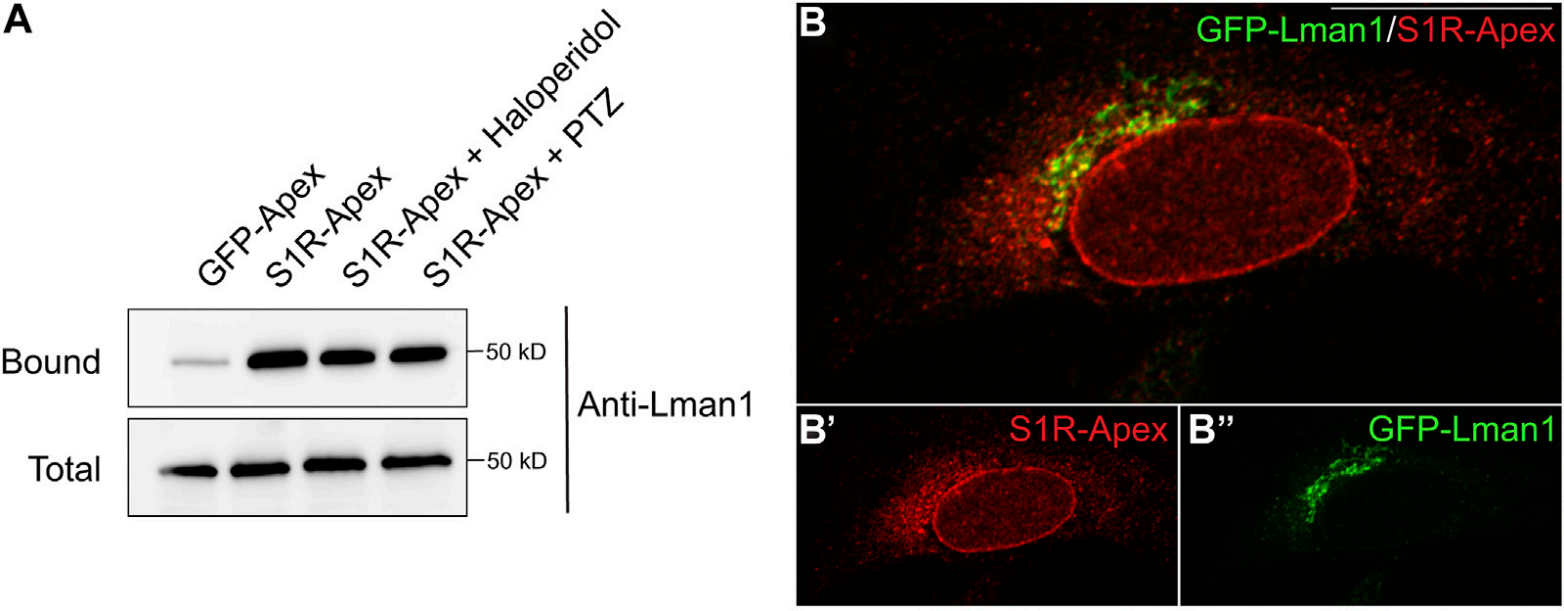
S1R biotinylates the ER-Golgi intermediate compartment component Lman1. **(A)** Biotinylated proteins were purified using streptavidin conjugated beads from HEK293T cells stably expressing either GFP-Apex or S1R-Apex that were untreated or treated with either Haloperidol or (+)-PTZ. The bound proteins were eluted and analyzed by Western blotting using an antibody against Lman1. S1R-Apex expressed in HEK293T cells biotinylates the ERGIC component Lman1. **(B)** HeLa cells stably expressing S1R-Apex were transfected with a plasmid expressing GFP-Lamn1 (green). The cells were fixed and processed for immunofluorescence using a V5 antibody (red). S1R-Apex co-localizes with GFP-Lman1 in the area of the ER-Golgi intermediate compartment.

Another category of proteins enriched in the S1R proximatome are proteins that localize to the ER-Golgi intermediate compartment (ERGIC) (Figure 2B). Proteins that localize at this site are involved in trafficking between the ER and Golgi compartments and often have a role in the secretory process (Appenzeller-Herzog and Hauri, 2006). The S1R proximatome contains several proteins that are secreted or localized on the plasma membrane such as Collagens, Integrins, and Low Density Lipoprotein Receptor (Supplementary Table S1). It is therefore possible that S1R plays an active role in the secretory process. In order to validate this finding, the binding experiment was repeated, and the pellets were analyzed by Western blotting using an antibody against Lman1, a marker protein of the ERGIC and a protein that was highly enriched in the S1R proteomics dataset (Supplementary Table S1) (Schindler et al., 1993). Lman1 was specifically biotinyated by S1R-Apex irrespective of the ligan bound state of receptor (Figure 4A). In addition, we observed significant co-localization between S1R and GFP-Lman1 (Figure 4B).

### Ligand dependent proximatome of S1R

Having established the S1R proximatome under native conditions, we next wished to determine how the proximatome would change when cells were treated with Haloperidol, an S1R antagonist, or with (+)-PTZ, an agonist of S1R (de Costa et al., 1989; Hayashi and Su, 2004; Vavers et al., 2019). For this experiment, HeLa cells expressing S1R-Apex were either untreated, treated with 25uM Haloperidol for 24 h, or treated with 20uM (+)-PTZ for 24 h. The localization of S1R was relatively unchanged under these treatment conditions (Supplementary Figures S2A–C). The treated cells were labeled, the biotinylated proteins were purified using streptavidin agarose and the bound proteins were analyzed using mass-spectrometry. As before, the entire experiment was done in triplicate.

**FIGURE 5:**
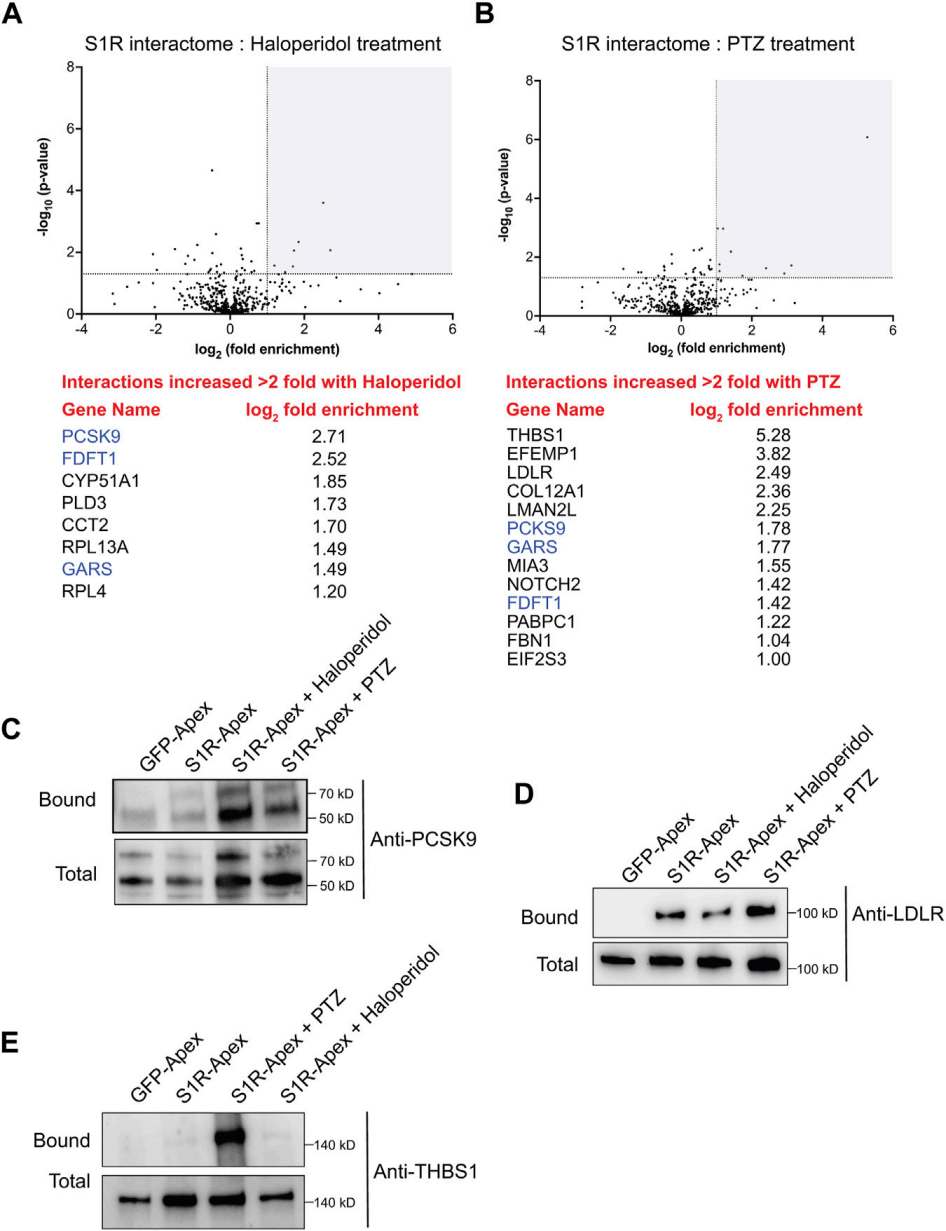
The ligand-dependent S1R proximatome. **(A)** A volcano plot comparing the proximatome of S1R-Apex under untreated conditions *versus* treatment with 25uM Haloperidol for 24 h. A line demarcating 2-fold enrichment and a *p*-value of 0.05 is shown. The grey shaded box indicates proteins that show at least 2-fold greater binding to S1R under Haloperidol treatment conditions with a *p*-value of at least 0.05. A list of these proteins is shown below the volcano plot. **(B)** A volcano plot comparing the proximatome of S1R-Apex under untreated conditions *versus* treatment with 20uM (+)-PTZ for 24 h. The layout is similar to panel **(A)**. The proteins in blue were shown to have an increased proximal association with S1R under both Haloperidol and (+)-PTZ treatment conditions. **(C–E)** Biotinylated proteins were purified from HeLa cells stably expressing either GFP-Apex or S1R-Apex that were untreated or treated with either Haloperidol or (+)-PTZ. The proteins were purified using streptavidin conjugated beads. The bound proteins were eluted and analyzed by Western blotting using antibodies against PCSK9 **(C)**, LDLR **(D)** or Thrombospondin1 **(E)**. Haloperidol treatment increased the proximal association between S1R and PCSK9, whereas (+)-PTZ treatment increases the proximal association between S1R LDLR and Thrombospondin1.

Somewhat surprisingly, treatment of cells with Haloperidol or (+)-PTZ did not change the biotinylation profile for the vast majority of proteins (Figures 5A, B, Supplementary Tables S2, S3). For this analysis, we considered proteins that were enriched at least two-fold (1.0 fold in the log2 scale) in the drug treated sample in comparison to the control and with a *p*-value of at least 0.05 as being significantly changed. Using these criteria, eight proteins displayed increased biotinylation by S1R-Apex in the presence of Haloperidol (Figure 5A) and thirteen proteins displayed increased biotinylation by S1R-Apex in the presence of (+)-PTZ (Figure 5B). Interestingly, even within this small set, three proteins were shared between the two drug-treated samples, FDFT1 which encodes Squalene synthase, PCKS9 which encodes Proprotein Convertase Subtilisin/Kexin Type 9, and GARS which encodes glycyl-tRNA-synthetase. Although Haloperidol is considered an antagonist and (+)-PTZ is considered an agonist of S1R, a recent structural study found that both compounds bind within the same binding pocket of S1R (Schmidt et al., 2018). Thus, in addition to unique structural changes that might be induced upon ligand binding, there might also be some common changes that are promoted by Haloperidol and (+)-PTZ.

For follow-up studies, we decided to focus our efforts primarily on PCSK9 and LDLR. PCSK9 (Proprotein Convertase Subtilisin/Kexin Type 9) displays increased biotinylation by S1R-Apex under conditions of Haloperidol treatment and to a lesser extent under PTZ treatment conditions, whereas LDLR (Low density lipoprotein receptor) is biotinylated more efficiently to S1R-Apex upon treatment with (+)-PTZ. Disease-causing variants in PCSK9 and loss of function mutations in LDLR are associated with familial hypercholesterolemia (Brown and Goldstein, 1986; Abifadel et al., 2003). PCSK9 is a secreted protein and extracellular PCSK9 binds to LDLR present on the cell surface. This results in endocytosis of LDLR and targeting of LDLR to the lysosome for degradation (Weinreich and Frishman, 2014). Increased turnover of LDLR results in high serum cholesterol levels (Cohen et al., 2006). Thus, both proteins play an important role in cholesterol metabolism. In addition, FDFT1, which encodes Squalene synthase, and CYP51A1, which encodes Lanosterol 14-alpha demethylase, are also a critical players in the cholesterol biosynthetic pathway (Shechter et al., 1992; Lorbek et al., 2012).

Cells expressing either GFP-Apex, S1R-Apex (untreated), or S1R-Apex treated with either Haloperidol or (+)-PTZ were labeled and the binding reaction was performed as before. Bound proteins were eluted and analyzed by Western blotting using an antibody against PCSK9 (Figure 5C) or LDLR (Figure 5D). Consistent with the proteomics results, treatment of cells with Haloperidol increased the amount of PCSK9 that was labeled and precipitated by S1R-Apex (Figure 5C). (+)-PTZ treatment also resulted in an enrichment of PCKS9 in the S1R-Apex bound sample. Similarly, treatment with (+)-PTZ increased the amount of LDLR that was labeled and precipitated in the S1R-Apex sample (Figure 5D). Another protein that also displayed an increased biotinylation by S1R-Apex in the presence of (+)-PTZ was Thrombospondin1 (THBS1), a finding that was also validated by Western blotting (Figures 5B, E).

### S1R regulates the cellular secretome

**FIGURE 6:**
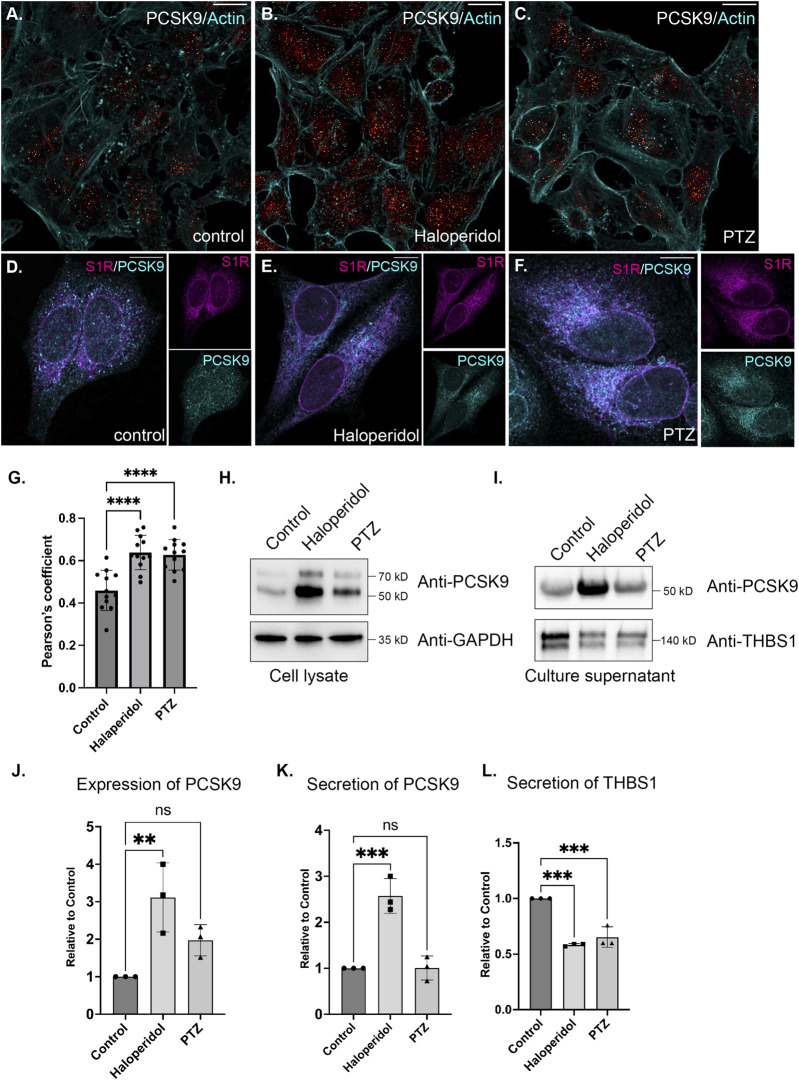
The effect of S1R ligand binding on PCSK9 and Thrombospondin1. **(A–C)** HeLa cells were untreated **(A)** or treated with either Haloperidol **(B)** or (+)-PTZ **(C)** for 24 h. The cells were then fixed and processed for immunofluorescence using an antibody against PCSK9. The cells were also counterstained using Alexa-633 conjugated Phalloidin to reveal the actin cytoskeleton (cyan). The signal for PCSK9 is depicted using a red to white range indicator, also known as a look-up-table (LUT). Low intensity pixels are shown in red and high intensity pixels are shown in white. The scale bar is 20 microns. **(D–F)** HeLa cells were untreated **(D)** or treated with either Haloperidol **(E)** or (+)-PTZ **(F)** for 24 h. The cells were then fixed and processed for immunofluorescence using antibodies against PCSK9 (cyan) and V5 (magenta). Individual channels as well as a merged image is shown. The scale bar is 20 microns. **(G)** Co-localization between S1R (V5) and PCKS9 was analyzed using Imaris 10. A Pearson’s coefficient is shown. Using this analysis method, perfect co-localization has a value of 1.0. S1R partially co-localizes with PCKS9 and the level of co-localizaiton is increased under conditions of ligand treatment. *n* = 12 images per condition. A one-way Anova was used for these analyses; ****p < 0.0001. **(H)** Lysates were prepared from HeLa cells that were either untreated or treated with either Haloperidol or (+)-PTZ. The lysates were analyzed by Western blotting using antibodies against PCKS9 and GAPDH. **(I)** HeLa cells were left untreated or were treated with either Haloperidol or (+)-PTZ for 24 h. For the last 4 h of this treatment, the media was replaced with serum free medium. The cells were then cultured for the final 4 h. The culture supernatant was collected, concentrated and analyzed by Western blotting using antibodies against either PCSK9 or Thrombospondin1. **(J)** The experiment in panel H was repeated in triplicate and the level of PCSK9 was quantified relative to the control untreated sample. A one-way Anova was used for these analyses; **p < 0.01, ns = not significant. Haloperidol treatment results in the upregulation of PCSK9 levels. **(K,L)** The experiment on panel I was repeated in triplicate and the level of secreted PCSK9 **(K)** and Thrombospondin1 **(L)** was quantified relative to the untreated sample. A one-way Anova was used for these analyses; ***p < 0.001, ns = not significant. Haloperidol treatment results in increased secretion of PCKS9. However, the sercretion of Thrombospondin1 is decreaed under these same conditions.

In order to follow up on these results, we examined the localization of PCSK9 in control cells or in cells treated with either Haloperidol or (+)-PTZ. PCSK9 was localized in small cytoplasmic foci. Although the pattern of localization was unchanged upon drug treatment, the level of PCKS9 was elevated upon treatment with Haloperidol and to a lesser extent with (+)-PTZ (Figures 6A–C). This result was confirmed using Western blot analysis (Figures 6H, J). Given the increased biotinylating of PCSK9 by S1R-Apex after drug treatment, we evaluated potential co-localizaiton between these two proteins. Partial co-localization was observed between PCSK9 and S1R-Apex under native conditions (Figures 6D, G). Moreover, the level of co-localizaiton was increased in cells treated with either Haloperidol or (+)-PTZ (Figures 6E–G).

PCSK9 is a secreted protein. We therefore monitored the secretion of PCSK9 under conditions of Haloperidol treatment. Culture supernatants were collected and analyzed by Western blotting. In comparison to untreated and (+)-PTZ treated cells, culture supernatant from Haloperidol treated cells contained significantly more PCSK9 (Figures 6I, K). In order to determine whether Haloperidol treatment increased global secretion we examined culture supernatants using an antibody against Thrombospondin1. In contrast to PCSK9, Thrombospondin1 was slightly decreased in culture supernatants from Haloperidol treated cells (Figures 6I, L). These findings suggest that Haloperidol does not globally affect protein secretion and that the effect on PCSK9 is likely to be specific. Interestingly, under these conditions, (+)-PTZ treatment also reduced the secretion of Thrombospondin1 (Figures 6I, L).

**FIGURE 7:**
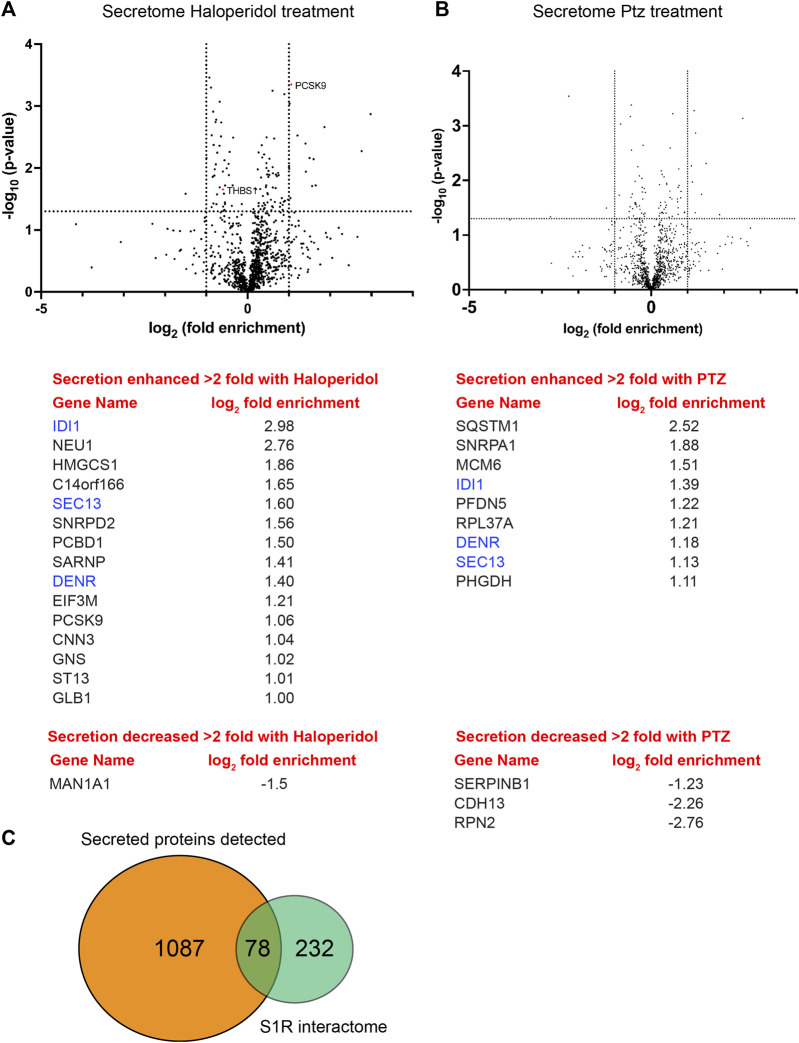
Global secretome analysis upon ligand treatment. **(A)** A volcano plot comparing the secretome between control cells *versus* cells treated with 25uM Haloperidol for 24 h. A line demarcating 2-fold enrichment (+1 and −1 on the x-axis) and a *p*-value of 0.05 is shown (1.3 on the y-axis). A list of proteins whose secretion is affected greater than two fold is shown below the volcano plot. **(B)** A volcano plot comparing the secretome between control cells *versus* cells treated with 20uM (+)-PTZ for 24 h. The layout is similar to panel **(A)**. The proteins in blue were shown to be affected in their secretion under both treatment conditions. **(C)** A pie chart showing the total number of secreted proteins, the number of proteins found to be biotinylated by S1R-Apex, and the overlap between the two categories.

We next compared the global secretome from control HeLa cells *versus* cells treated with either Haloperidol or (+)-PTZ. For this experiment, cells were cultured in serum containing media for 20 h, after which time the media was removed and replaced with serum free media for 4 h. As with previous experiments, cells were either untreated or treated with Haloperidol or (+)-PTZ for 24 h. Conditioned medium was collected, concentrated, and analyzed by mass spectrometry. A total of 1,087 proteins were detected in culture supernatant (Supplementary Table S4). The vast majority of these proteins were unchanged in their secretion upon treatment with either Haloperidol or (+)-PTZ. However, 15 proteins, including PCSK9, showed a greater than 2 fold increase in their secretion upon Haloperidol treatment (Figure 7A; Supplementary Table S5). Only 1 protein was reduced in its secretion more than 2 fold upon Haloperidol treatment (Figure 7A; Supplementary Table S5). Consistent with our prior observation via Western blot analysis (Figure 6I), we detected a statistically significant decrease in the level of Thrombospondin1 upon Haloperidol treatment (Figure 7A). However, the difference by mass spectrometry analysis of conditioned media was less than 2 fold (Supplementary Table S5). In (+)-PTZ treated samples, 9 proteins showed a greater than 2 fold increase in secretion (Figure 7B, Supplementary Table S6). Interestingly, 3 of these proteins also displayed increased secretion under Haloperidol treatment conditions (Figures 7A, B, blue font). (+)-PTZ treatment resulted in a greater than 2 fold decrease in the secretion of 3 proteins (Figure 7B; Supplementary Table S6). Collectively, these results suggest that the ligand bound state of S1R can specifically alter the cellular secretome.

### S1R, LDLR and cholesterol

**FIGURE 8:**
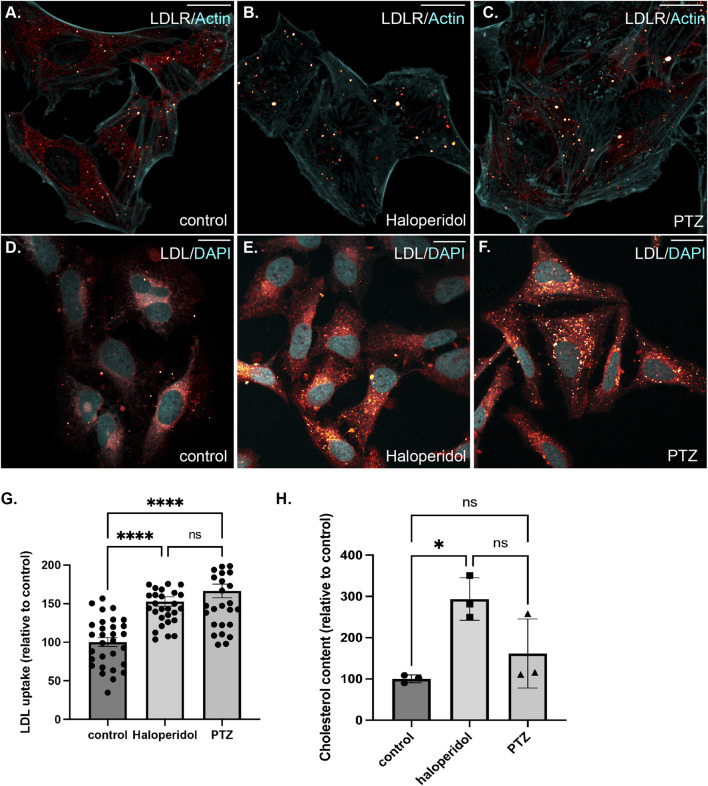
The effect of S1R ligand binding on LDLR, LDL uptake and Cholesterol. **(A–C)** HeLa cells were untreated **(A)** or treated with either Haloperidol **(B)** or (+)-PTZ **(C)** for 24 h. The cells were then fixed and processed for immunofluorescence using an antibody against LDLR. The cells were also counterstained using Alexa-633 conjugated Phalloidin to reveal the actin cytoskeleton (cyan). The signal for LDLR is depicted using a red to white look-up-table (LUT). Haloperidol treatment causes LDLR to accumulate within large intracellular puncta. A similar but milder phenotype was observed in (+)-PTZ treated cells. The scale bar is 20 microns. **(D–F)** HeLa cells were untreated (G) or treated with either Haloperidol (H) or (+)-PTZ (I) for 24 h. The cells were then incubated with fluorescently labeled LDL. After 2 h of incubation, the cells were fixed and counterstained with DAPI to reveal nuclei (cyan). The signal for LDL is shown using the red to white LUT. The scale bar is 20 microns. **(G)** The LDL assay was repeated in triplicate. The fluorescence intensity per cell was calculated for twenty frames for each treatment condition. This was used to determine the mean intensity for each treatment. The LDL uptake value for the Haloperidol and (+)-PTZ treated samples are reported relative to the control sample. A one-way Anova was used for these analyses; ****p < 0.0001, ns = not significant. The ability of cells to endocytose LDL is increased upon treatment with either Haloperidol or (+)-PTZ. **(H)** Cells were left untreated or were treated for 48 h with either Haloperidol or (+)-PTZ. Lipids were extracted from the cells and the cholesertol content was measured using the Amplex Red kit. The mean value for each condition was determined. The mean cholesterol content in the treated samples is reported relative to the mean of the untreated sample. In comparison to the control, Haloperidol treated cells contain a higher cholesterol content. Although a trend towards higher cholesterol content was also observed in the (+)-PTZ treated cells, this result was not statistically significant. A one-way Anova was used for these analyses. *p < 0.05, ns = not significant.

We next turned our attention to LDLR. In control cells, LDLR was diffusely localized within the cytoplasm with occasional localization within small puncta (Figure 8A). Interestingly, Haloperidol treatment caused a dramatic relocalization of LDLR to large intracellular foci (Figure 8B). A similar, but milder phenotype was observed in (+)-PTZ treated cells (Figure 8C). Thus, although Haloperidol and (+)-PTZ do not affect the cellular level of LDLR, both drugs affect the intracellular localization of the receptor. Co-staining cells with antibodies recognizing LDLR and S1R-Apex indicated a partial co-localization between both proteins (Supplementary Figures S2D–G). However, unlike with PCKS9, the level of co-localizaton between S1R and LDLR was unchanged upon treatment of cells with either Haloperidol or (+)-PTZ (Supplementary Figures S2E–G).

LDLR is present on the cell surface where it binds to extracellular LDL resulting in its endocytosis. Internalized LDL is trafficked through the endocytic pathway to the lysosome. Lysosomal degradation of LDL releases cholesterol, which can then be used by the cell (Goldstein and Brown, 1977; Brown and Goldstein, 1997). We therefore examined the ability of cells that were treated with Haloperidol or (+)-PTZ to endocytose labelled LDL. In comparison to untreated cells, both (+)-PTZ and Haloperidol treatment resulted in increased LDL uptake (Figures 8G–J). This result was somewhat surprising for Haloperidol treated cells. Higher levels of PSCK9 are generally thought to result in lower LDLR levels and therefore reduced LDL uptake. Possible scenarios that might explain this unanticipated result are discussed below. We next measured the cholesterol content of cells treated with either (+)-PTZ or Haloperidol. In comparison to control, the cholesterol content was higher in cells treated with Haloperidol (Figure 8H). A similar trend of higher cholesterol content was also observed in (+)-PTZ treated cells. However, under the conditions of this experiment, the difference was not statistically significant (Figure 8H).

Collectively, our findings reveal that the S1R proximatome consists of over 200 proteins. Ligand dependent changes involve proteins that are involved in cholesterol metabolism as well as proteins that are secreted into the extracellular space. The therapeutic benefit of S1R activation likely involves these and other proximatome changes.

## Discussion

We report here use of a cell line-based proximity labeling assay to probe the S1R proximatome under native conditions and in a ligand-dependent manner. This methodology, combined with quantitative mass spectrometry-based proteomics, identified over 200 S1R-proximal proteins. Proteins identified in this manner do not necessarily represent direct interaction partners. However, given the limited labeling radius of Apex2, it is likely that many of the identified proteins are present in a complex with S1R. However, additional experiments will be required to determine whether these proximal associations represent true biochemical interactions. The advantage of proximity biotin labeling is that weak interactions, which might otherwise be missed in a classical purification approach, might be identified using this strategy. Consistent with this notion, although we were able to easily biotinylate and purify Bip, Calnexin, and Protein Disulfide Isomerase (PDI) using S1R-Apex, these proteins did not efficiently co-immunoprecipitate with S1R-Apex (Supplementary Figure S1B and data not shown).

The recently published crystal structure of S1R shows that it consists of a single transmembrane segment with a short cytoplasmic tail and a large luminal ligand-binding domain (Schmidt et al., 2016). High resolution electron microscopy studies using an S1R-GFP-APEX2 fusion protein are consistent with luminal localization of the ligand-binding S1R C-terminus (Mavlyutov et al., 2017). Importantly, our experiments also involved tagging S1R with Apex on the C-terminus of the protein. Our results indicate that the S1R proximatome consists mostly of proteins residing within the ER lumen, the ER membrane, or within the secretory pathway. This provides functional evidence for a luminal localization of the ligand-binding domain of S1R, and is consistent with structural studies.

Previous studies indicate that S1R can influence the cellular function of many proteins (Su et al., 2016; Schmidt and Kruse, 2019). A proposed mechanism for S1R’s multiple intracellular effects has been through direct protein interactions, and S1R has been termed a ligand-operated molecular chaperone (Hayashi and Su, 2007; Su et al., 2010). In support of this model, experiments performed in Chinese Hamster Ovary (CHO) cells indicate that S1R forms a complex with the chaperone protein, BiP, which is known to play a central role in protein folding and quality control (Hayashi and Su, 2007). Studies also showed that activation of S1R by the agonist (+)PTZ, led to dissociation of S1R from BiP. Release from BiP was proposed to trigger S1R’s multiple interactions with client proteins (Hayashi and Su, 2003; Hayashi and Su, 2007).

Our results (Figure 2) suggest a proximal association between S1R and BiP, consistent with previous studies. However, we did not observe a difference in the biotinylation status of Bip by S1R-Apex when cells were exposed to either Haloperidol or (+)-PTZ (Figure 5). In addition, in native condition HeLa cells (without antagonist or agonist treatment), S1R-Apex biotinylates not only Bip, but also hundreds of other proteins (Figure 2). Furthermore, the number of biotinylated proteins does not substantially increase with exposure to ligands (Figure 5). In contrast to previous studies, our results offer a comprehensive and quantitative view of the S1R proximatome, and future work should address how S1R-protein interactions vary dependent on cell type analyzed, ligand specificity and exposure time as well as cell culture conditions.

Our data do show a large, robust and reproducible S1R-Apex proximatome, consistent with the paradigm of S1R-mediated chaperone function. Our studies indicate a proximal association between S1R and the ER translocation complex. We have validated the biotinylation of Sec61alpha by S1R-Apex and show that this proximal association is sensitive to protein import into the ER. Overall, consistent with previous work, our studies support ER-localized chaperone functionalities as central to the mechanism of S1R’s pleotropic effects.

A recent study by Zhemkov and colleagues also reported on the S1R proximatome in native HeLa cells (Zhemkov et al., 2021a). Our results are broadly consistent with theirs. For instance, numerous proteins such as PDI, Calnexin, Integrin, and PCKS9 were identified as S1R proximal proteins in both studies. However, the proximatomes were not completely overlapping. The study by Zhemkov et al., used a Tetracyline inducible expression system for expressing S1R-Apex, whereas in this study S1R-Apex was expressed constitutively after integration at the *AAVS1* safe harbor locus. In addition, the binding control used in the Zhemkov et al. study corresponeded to uninduced cells (i.e., no expression of S1R-Apex), whereas in the current study, cells expressing GFP-Apex were used as the control.

In addition to evaluating the native S1R proximatome, our study assessed proximatome changes in cells treated with S1R ligands. For these studies, we used two classic ligands with high affinity for S1R. Traditionally, these chemicals have been classified as S1R agonists or antagonists based on their ability to recapitulate the effects of genetic knockout (KO) or knockdown of S1R. For example, ligands that mimic S1R genetic KO are considered antagonists (Nguyen et al., 2015). S1R KO animals are viable and fertile, but show signs and symptoms consistent with exacerbation of age-associated neurodegenerative disorders (Sabino et al., 2009). The KO studies collectively highlight the innate prosurvival and neuroprotective properties of S1R activity. Therefore, S1R ligands that promote cellular survival and neuroprotection are generally considered agonists.

The two ligands in our studies, (+)-Pentazocine, (+)-PTZ, an agonist, and Haloperidol, an antagonist, were each used in the original structural analyses that characterized S1R ligand recognition (Schmidt et al., 2018). PTZ is an FDA-approved analgesic that exists as a racemic mixture of dextro-(+) and levo-(−) isomers. The levo-(−) isomer shows high affinity for opioid receptors and the dextro-(+) isomer (referred to in this study as (+)-PTZ) shows high affinity for S1R. Effects of (+)-PTZ are generally neuroprotective and pro-survival, and it is considered the prototypical S1R agonist (Walker et al., 1990). Haloperidol, the S1R antagonist used in this study, is a first generation (typical), FDA-approved, antipsychotic (Shader and Shader, 1994). It mitigates positive symptoms of schizophrenia by blocking dopamine D2 receptors in the brain. However, Haloperidol is not selective for D2 receptors, and also has high affinity for S1Rs. As such it has been used extensively in cellular, molecular and biochemical studies of ligand-dependent S1R effects (Mei and Pasternak, 2007; Bai et al., 2017).

Our results provide a unique and important contribution to knowledge of S1R biology. Results are notable for enrichment of proteins integral to secretion and extracellular matrix formation as well as cholesterol biosynthesis and metabolism (Figure 5). Consistent with the latter result, previous studies have shown that S1R interacts with cholesterol and have implicated S1R in lipid metabolism and transport (Palmer et al., 2007; Hayashi and Su, 2010; Zhemkov et al., 2021b). However, the mechanisms that underlie these interactions are not well understood.

Our results indicate that under conditions of cellular exposure to Haloperidol, the proximal association between S1R-Apex and Proprotein convertase subtilisin/kexin type 9 (PCSK9) was increased. In addition, exposure to (+)-PTZ also increased the proximal association between S1R and PCSK9 and between S1R and the low density lipoprotein receptor (LDLR) (Figure 5). PCSK9 and LDLR are known to interact with each other and both are medically important because mutations in both genes are associated with familial hypercholesterolemia (Abifadel et al., 2003; Abifadel et al., 2009a; Abifadel et al., 2009b; Abifadel et al., 2012). LDLR, situated on cellular membranes, binds to and mediates endocytosis of cholesterol-rich LDL from the extracellular space. The endocytosed LDL is eventually broken down and cholesterol is released for use by the cell. Interaction between PCSK9 and LDLR has been shown to lead to lysosome-mediated destruction of LDLR and therefore to decreased levels of LDLR at the cellular surface (Brown and Goldstein, 1986). In general, a decrease in cell surface LDLR levels increases concentration of systemically circulating LDL-cholesterol (Brown and Goldstein, 1986). Therefore, inhibition of PCSK9 is clinically useful for treatment of hypercholesterolemia, and inhibitors of PCSK9 have been FDA-approved as cholesterol-lowering therapeutics (Weinreich and Frishman, 2014).

Our findings indicate that Haloperidol treatment not only increases the proximal association between S1R-Apex and PCSK9, but also leads to increased levels of intracellular and extracellular (secreted) PCSK9 (Figures 6, 7). Somewhat surprisingly, LDLR levels were unaffected by Haloperidol treatment, at least under the conditions of our experimental setup (Figure 5D). This finding was unexpected given the documented role of PCSK9 in mediating turnover of LDLR. However, the organ that is mostly responsible for producing and secreting PCSK9 is the liver. Secreted PCSK9 can then alter the level of LDLR in distal tissues, a scenario that is different from the cell culture setup used in our experiments. Another possibility is that treatment with Haloperidol for an extended duration might be required to observe a PCSK9-induced reduction in LDLR levels. Although Haloperidol treatment did not affect the level of LDLR, the intracellular localization of LDLR was dramatically altered. Instead of mostly diffuse cytoplasmic staining, LDLR was localized to large puncta in Haloperidol treated cells (Figure 8). At present, the mechanism that causes this relocalization of intracellular LDLR is unknown.

In addition to exploring effects of Haloperidol, a known S1R antagonist, on the S1R proximatome, we also evaluated the effects of the high affinity S1R agonist, (+)-Pentazocine. Most of the proximatome changes induced by (+)-PTZ correspond to secreted and extracellular matrix proteins such as Thrombospondin1 (THBS1), Collagen12 (COL12A1) and Fibulin 1 and 3 (EFEMP1 and FBN1 respectively) (Figure 5). For this set, we validated the biotinylation between S1R-Apex and Thrombospondin1 (Figure 5E). In addition, proteins involved in cholesterol metabolism and homeostasis such as squalene synthase (FDFT1), PCKS9, and LDLR were also enriched in this dataset. Consistent with our proteomics results, (+)-PTZ treatment resulted in increased biotinylation of LDLR by S1R-Apex (Figure 5D). The intracellular localization of LDLR was also altered upon (+)-PTZ treatment, but to a lesser degree than when cells were treated with Haloperidol (Figure 7).

One common theme between (+)-PTZ and Haloperidol treatment appears to involve cholesterol metabolism. Both ligands affect the localization of intracellular LDLR and treatment with both compounds promoted the cellular uptake of labeled LDL (Figure 7). Consistent with a role for S1R in cholesterol metabolism, treatment with Haloperidol increased the total cellular level of cholesterol (Figure 8H). A similar trend was observed in (+)-PTZ treated cells. However, the difference was not statistically significant. The effects of S1R ligand binding on cholesterol metabolism is medically important because medications with S1R affinity are already in widespread use (Gordon et al., 2020; Ye et al., 2020; Maurice, 2021; Reis et al., 2022).

Another common theme that emerges from our studies is the involvement of S1R in modulation of protein secretion. Proteins destined for secretion are known to move from ER exit sites through the ER-Golgi intermediate compartment (ERGIC) (Appenzeller-Herzog and Hauri, 2006). As shown in Figure 2B, components of the ERGIC are enriched in the S1R proximatome. This finding was validated by Western blot analysis of pellets using an antibody against Lman1, a marker protein of the ERGIC, and a protein that was highly enriched in the S1R proximatome (Figure 4A and Supplementary Table S1) (Hauri et al., 2000; Fu et al., 2019). Furthermore, a globlal secretome analysis identified a total of 1,087 proteins in HeLa cell conditioned media. Of these, 78 proteins were also detected in the S1R proximatome (Figure 7C; Supplementary Table S7). Interestingly, the ligand-bound state of S1R correlated with increased or decreased secretion of a subset of these proteins (Figure 7). Our results are therefore consistent with previous studies that implicate S1R as an important regulator of the cellular secretome. For example, stimulation of S1R with (+)-PTZ leads to increased astrocytic release of brain-derived neurotrophic factor (BDNF), a neurotrophin that supports neuronal growth and survival (Mysona et al., 2018). In addition, S1R has been shown to regulate levels of several other proteins that are processed and transported through the cellular secretory pathway (Maher et al., 2018; Lopez et al., 2019).

Collectively, our findings indicate that the S1R-Apex proximatome contains a large subset of proteins. Somewhat unexpectedly, the S1R-Apex proximatome was not significantly altered upon treating cells with either a classical antagonist or agonist. It should be noted, however, that our analysis only considered proteins showing a greater than two-fold, ligand-dependent biotinylation change as significant. In an organismal setting, proteins displaying more subtle changes upon ligand binding might nevertheless elicit physiological effects. Furthermore, the time scale of ligand incubation might affect the degree and nature of proximatome changes. Our proximatome experiments were performed after 24 h of ligand treatment. Additional time points might have revealed different changes. A final consideration is that the S1R proximatome might be different in different cell types and tissues. Despite these caveats, S1R agonists and antagonists are under consideration for an ever-widening spectrum of pathologies ranging from COVID-19 treatment to cancer diagnosis, chronic pain remedies, and neurodegenerative disease therapeutics (Happy et al., 2015; Das et al., 2016; Vavers et al., 2019; Vela, 2020). Our results are therefore critically relevant to a broad range of translational outcomes.

## Experimental procedures

### DNA constructs cell lines

The S1R-Apex construct was generated by cloning a gene synthesized product containing the cDNA sequence for mouse S1R into the pCDNA3_Sec61B-V5-APEX2 vector (Addgene plasmid 83411) (Lee et al., 2016). Gene synthesized products were obtained from Genewiz. Gibson assembly was used to replace the Sec61B cDNA in this vector with the sequence for S1R. A similar strategy was used to construct the GFP-Apex plasmid. These constructs were then subcloned into the pDONR221 Gateway vector (Life Technologies) and moved into the pAAVS1-P-CAG-DEST vector (Addgene plasmid 80490) (Oceguera-Yanez et al., 2016). This vector enables insertion into the AAVS1 safe harbor locus present in human cell lines and also enables selection of properly integrated cells using Puromycin. All final constructs were verified by sequencing prior to use. The S1R-Apex and GFP-Apex plasmids were co-transfected into either HeLa cells (ATCC; CCL-2) or HEK293T cells (ATCC; CRL-3216) along with pXAT2 (Addgene plasmid 80494) (Oceguera-Yanez et al., 2016). The pXAT2 vector expresses the guide RNA for the AAVS1 locus. Effectene (Qiagen) was used as the transfection reagent. Two days after transfection, stable cells were selected using 0.5 ug/mL (HeLa) or 1 ug/mL (HEK293T) of Puromycin (Millipore-Sigma). The plasmid expressing GFP-Lman1 was obtained from Addgene (Addgene plasmid 166942) (Ward et al., 2001).

### Immunofluorescence

Immunofluorescence was performed as previously described (Hicks et al., 2015). In brief, cells were fixed in 4% formaldehyde (Pierce, ThermoFisher) for 5 min at room temperature. The cells were permeabilized by washing in PBST (PBS +0.1% Triton X-100). The primary antibody was incubated in blocking buffer (PBS +5% Normal goat serum) overnight at 4°C. Next, the samples were washed three times in PBST and incubated for 1 h at room temperature with the fluorescent secondary antibody (Goat anti-mouse or Goat anti-rabbit antibodies conjugated with either Alexa488 or Alexa555, 1:400 dilution, Life technologies) in the same blocking buffer. The samples were then washed four times with PBST. In order to visualize nuclei, the cells were stained with DAPI. For visualizing the actin cytoskeleton Alexa633 conjugated Phalloidin was used (Life technologies; 1:400). For detecting biotinylated proteins, the samples were incubated with Streptavidin Alexa647 (Life technologies; 1:1,200). The Strep674 was added to the sample at the same time as the secondary antibody. The cells were mounted onto slides using Prolong Diamond (Life technologies).

### Antibodies

The following antibodies were used: V5 was used to visualize GFP-Apex and S1R-Apex (ThermoFisher; 1:10,000 for western, 1:1,000 for immunofluorescence); Bip (Cell Signaling; 1:1,000 for western); PDI (Cell Signaling; 1:1,000 for western); Calnexin (Cell Signaling; 1:1,000 for western); Sec61alpha (Santa Cruz; 1:100 for western); Sigma1 Receptor (1:500 for western) (Ola et al., 2002); Lman1 (Abcam; 1:1,000 for western); PCSK9 (Abcam; 1:3,000 for western, 1:300 for immunofluorescence); LDLR (Novus biologicals; 1:1,000 for western, 1:100 for immunofluorescence); Thrombospondin (Abcam; 1:250 for western); Gapdh (Santa Cruz; 1:2000 for western).

### Protein purification

Biotin labeling was performed based on a previously published protocol (Hung et al., 2016). HeLa cells stably expressing either GFP-Apex or S1R-Apex were plated on 100 mm culture dishes in DMEM (ThermoFisher, Waltham, MA) supplemented with 10% FBS (Atlanta Biologicals, Atlanta, GA) and 1% penicillin/streptomycin (ThermoFisher). For each replicate three 100 mm dishes of cells expressing GFP-Apex and three 100 mm dishes of cells expressing S1R-Apex were used. This corresponds to approximately 2.5 mg of total protein from each sample. A total of three biological replicates were processed for proteomic analysis. The following day after seeding, the cells were either untreated (GFP-Apex and S1R-Apex) or treated (S1R-Apex) with 25 µM Haloperidol (Tocris, Minneapolis, MN) or 20 µM (+)-PTZ (Millipore-Sigma, St. Louis, MO) for 24 h. Next, 500 µM Biotin-Phenol (Millipore-Sigma) in complete medium was added to the cells for 30 min at 37°C. After this incubation, H_2_O_2_ (Millipore-Sigma) was added to the cell to a final concentration of 1 mM. The cells were incubated for 1 min at room temperature. This solution was then removed, and the cells were washed three times in a quench solution (10 mM sodium ascorbate (Millipore-Sigma), 5 mM Trolox (Millipore-Sigma) and 10 mM sodium azide (Millipore-Sigma) in Dulbecco’s PBS (ThermoFisher). Next, the cells were harvested and pelleted by centrifugation. The supernatant was removed, and the cells were stored at −80°C until use. The cells were lysed in RIPA buffer (50 mM Tris-Cl pH 7.5, 150 mM NaCl, 1% NP40, and 1 mM EDTA) containing 0.2% SDS. The lysates were centrifuged at 10,000 g for 5 min at 4°C. Next, the biotinylated proteins were purified by incubating the lysates with High Capacity Streptavidin Agarose beads (Pierce, ThermoFisher) overnight at 4°C. The next day, the beads were washed three times with RIPA buffer, three times with 1% SDS, three times with RIPA buffer, three times with high salt RIPA buffer (50 mM Tris-Cl pH 7.5, 1M NaCl, 1% NP40, and 1 mM EDTA), three times with RIPA buffer, and finally with four PBS washes. The beads then re-suspended in PBS and processed for proteomics.

### Mass spectrometry

The beads with bound proteins were reduced with dithiothreitol, alkylated using iodoacetamide in 8M urea denaturation buffer (50 mM Tris-HCl, pH 8) and digested overnight in 50 mM ammonium bicarbonate using trypsin (ThermoFisher) at 37°C. Digested peptides were cleaned using a C18 spin column (Harvard Apparatus) and then lyophilized. Peptide digests were analyzed on an Orbitrap Fusion tribrid mass spectrometer (ThermoFisher) coupled with an Ultimate 3,000 nano-UPLC system (ThermoFisher). Two microliters of reconstituted peptide was first trapped and washed on a Pepmap100 C18 trap (5 um, 0.3 × 5 mm) at 20 ul/min using 2% acetonitrile in water (with 0.1% formic acid) for 10 min and then separated on a Pepman 100 RSLC C18 column (2.0 um, 75-μm × 150-mm) using a gradient of 2%–40% acetonitrile with 0.1% formic acid over 40 min at a flow rate of 300 nL/min and a column temperature of 40°C. Samples were analyzed by data-dependent acquisition in positive mode using Orbitrap MS analyzer for precursor scan at 120,000 FWHM from 300 to 1,500 m/z and ion-trap MS analyzer for MS/MS scans at top speed mode (3-s cycle time). Collision-induced dissociation (CID) was used as fragmentation method. Label-free quantification analysis was adapted from a published procedure (Seyfried et al., 2017). Spectra were searched using the search engine Andromeda, integrated into MaxQuant (version 1.6.15.0), against Human Uniprot/Swiss-Prot database (20,379 target sequences) (uniprot-human-swissprot-Feb2020. fasta). Methionine oxidation (+15.9949 Da), asparagine and glutamine deamidation (+0.9840 Da), and protein N-terminal acetylation (+42.0106 Da) were variable modifications (up to 5 allowed per peptide); cysteine was assigned as a fixed carbamidomethyl modification (+57.0215 Da). Only fully tryptic peptides were considered with up to 2 missed cleavages in the database search. A precursor mass tolerance of ±20 ppm was applied prior to mass accuracy calibration and ±4.5 ppm after internal MaxQuant calibration. Other search settings included a maximum peptide mass of 6,000 Da, a minimum peptide length of 6 residues, 0.05 Da tolerance for orbitrap and 0.6 Da tolerance for ion trap MS/MS scans. The false discovery rate (FDR) for peptide spectral matches, proteins, and site decoy fraction were all set to 1 percent. Quantification settings were as follows: re-quantify with a second peak finding attempt after protein identification has completed; match MS1 peaks between runs; a 0.7 min retention time match window was used after an alignment function was found with a 20-min RT search space. Quantitation of proteins was performed using summed peptide intensities given by MaxQuant. The quantitation method only considered razor plus unique peptides for protein level quantitation.

For the secretome analysis, in solution digestion of proteins was performed using a published protocol (Soucek et al., 2016). After BCA assay, conditioned media samples were normalized with the digestion buffer (50 mM NH_4_HCO_3_) to a concentration of 1 mg/mL 50 μg protein was then treated with 5 mM dithiothreitol (DTT) at room temperature for 30 min, followed by 10 mM iodoacetimide (IAA) at room temperature for 30 min in the dark. Proteins were digested with 2 µg of lysyl endopeptidase (Wako) at room temperature overnight and were further digested overnight with 2 µg trypsin (Promega) at room temperature. The resulting peptides were desalted using an HLB column (Waters) and were dried under vacuum. The data acquisition by LC-MS/MS was adapted from a published procedure (Seyfried et al., 2017). Derived peptides were resuspended in the loading buffer (0.1% trifluoroacetic acid, TFA) and were separated on a Water’s Charged Surface Hybrid (CSH) column (150 µm internal diameter (ID) x 15 cm; particle size: 1.7 µm). The samples were run on an EVOSEP liquid chromatography system using the 15 samples per day preset gradient (88 min) and were monitored on a Q-Exactive Plus Hybrid Quadrupole-Orbitrap Mass Spectrometer (ThermoFisher Scientific). The mass spectrometer cycle was programmed to collect one full MS scan followed by 20 data dependent MS/MS scans. The MS scans (400–1,600 m/z range, 3 × 10^6^ AGC target, 100 m maximum ion time) were collected at a resolution of 70,000 at m/z 200 in profile mode. The HCD MS/MS spectra (1.6 m/z isolation width, 28% collision energy, 1 × 10^5^ AGC target, 100 m maximum ion time) were acquired at a resolution of 17,500 at m/z 200. Dynamic exclusion was set to exclude previously sequenced precursor ions for 30 s. Precursor ions with +1, and +7, +8 or higher charge states were excluded from sequencing. Label-free quantification analysis was adapted from a published procedure (Seyfried et al., 2017). Spectra were searched using the search engine Andromeda, integrated into MaxQuant, against 2020 Uniprot/Swiss-Prot human database (20,379 target sequences). Methionine oxidation (+15.9949 Da), asparagine and glutamine deamidation (+0.9840 Da), and protein N-terminal acetylation (+42.0106 Da) were variable modifications (up to 5 allowed per peptide); cysteine was assigned as a fixed carbamidomethyl modification (+57.0215 Da). Only fully tryptic peptides were considered with up to 2 missed cleavages in the database search. A precursor mass tolerance of ±20 ppm was applied prior to mass accuracy calibration and ±4.5 ppm after internal MaxQuant calibration. Other search settings included a maximum peptide mass of 6,000 Da, a minimum peptide length of 6 residues, 0.05 Da tolerance for orbitrap and 0.6 Da tolerance for ion trap MS/MS scans. The false discovery rate (FDR) for peptide spectral matches, proteins, and site decoy fraction were all set to 1 percent. Quantification settings were as follows: re-quantify with a second peak finding attempt after protein identification has completed; match MS1 peaks between runs; a 0.7 min retention time match window was used after an alignment function was found with a 20-min RT search space. Quantitation of proteins was performed using summed peptide intensities given by MaxQuant. The quantitation method only considered razor plus unique peptides for protein level quantitation.

### Co-immunoprecipitation

Lysates were prepared from HeLa cells stably expressiong S1R-Apex using a 0.2% CHAPS buffer (0.2% CHAPS, 50 mM Tris-Cl pH 7.5, 150 mM NaCl, and 1 mM EDTA). 1000ug of total protein was used in each immunoprecipitation experiment. Anti-HA antibody (Santa Cruz Biotechnology) was used as a control and anti-V5 antibody (ThermoFisher) was used to precipitate S1R-Apex. The respective antibodies were incubated with the lysates for 1 h at 4°C. Next, the antibody complexes were pufiried using protein-A agarose beads (Pierce) at 4°C for 1 h. The beads were washed four times with 800 ul of 0.2% CHAPS buffer. The bounds proteins were eluted from the beads by boiling in Laemmli buffer and analyzed by Western blotting using the indicated antibodies.

### Western blotting

For the validation experiments using Western blotting, the same procedure was followed using ¼ the number of cells. 10 ul of whole cell lysate was collected prior to the binding to run as the total fraction. The same binding and wash steps were used. The bound proteins were eluted by boiling in Laemmli gel loading buffer. Proteins were separated by electrophoresis on a 4%–15% SDS-polyacrylamide gel (BioRad) and then transferred to a nitrocellulose membrane (ThermoFisher). The membrane was blocked with 5% nonfat milk in Tris-buffered saline-0.05% Tween 20 (TBST) for 1 h at room temperature, then incubated overnight at 4°C with primary antibodies. After three washes in TBST, the membrane was incubated for 1 h using an appropriate Horseradish Peroxidase (HRP)-conjugated secondary antibody (Santa Cruz Biotechnology) at room temperature. Proteins were visualized by incubating with a SuperSignal West Pico chemiluminescent substrate (ThermoFisher). A BioRad ChemidocXP was used to visualize and quantify Western blot signal.

### Microscopy

Images were captured on either a Zeiss LSM 780 inverted confocal microscope or an inverted Leica Stellaris confocal microscope located within the Augusta University Cell and Tissue imaging core.

### LDL uptake assay

HeLa cells were seeded onto glass coverslips. After the cells adhered, they were either left untreated or treated with 25 µM Haloperidol or 20 µM PTZ for a total of 24 h. The LDL uptake assay (Image-iT Low Density Lipoprotein Uptake kit, ThermoFisher) was performed as per the manufacturer’s instructions. Briefly, for the last 18 h of drug treatment, the medium was replaced with serum free DMEM containing 0.3% BSA, and then incubated with 10 ug/mL of labeled LDL at 37°C for 2 h. The cells were then washed twice with PBS and fixed with 4% formaldehyde (Pierce) for 5 min at room temperature. The cells were counterstained with DAPI. The coverslips were mounted onto slides using Fluoroshield (Millipore-Sigma). Cells were imaged for quantification using a Zeiss Axio Imager D2 microscope equipped with Zeiss Zen23pro software and a high-resolution camera. For high resolution images, cells were imaged on a Zeiss 780 inverted confocal microscope.

### Quantificaiton of cellular cholesterol content

The cholesterol assay (Life technologies) was performed per the manufacture’s instruction. Breifly, cholesterol-containing samples were prepared by extracting cholesterol/lipid from each 100 mm dish of Hela cells using chloroform-methanol-0.73% NaCl (2:1:0.8) solvent. The samples were then mixed with a working solution of 300 μM Amplex Red reagent containing 2 U/mL HRP, 2 U/mL cholesterol oxidase, and 0.2 U/mL cholesterol esterase, and then incubated at 37°C for 30 min. Flurescence was measured in a fluorescence microplate reader using an excitation filter in the range of 530–560 nm and an emission filter for detection at 590 nm.

### Software

Proteins levels were quantified using the ImageLab software (BioRad). The co-localizaiton analysis was performed using the co-loc module of Imaris 10.0. Images were processed for presentation using Fiji, Adobe Photoshop, and Adobe Illustrator. Graphs and volcano plots were assembled using Graphpad Prism9. Statistical analysis were also performed using Graphpad Prism.

## Data Availability

The original contributions presented in the study are included in the article/Supplementary Material, further inquiries can be directed to the corresponding authors.

## Supplementary material

SUPPLEMENTAL FIGURE S1
**(A)** HeLa cells stably expressing GFP-Apex were fixed and processed for immunofluorescence using a V5 antibody (green). The cells were also incubated with Streptavidin-647 (red) to reveal the localization of biotinylated proteins and were counterstained using DAPI (cyan). Biotinyalted proteins are observed in the nucleus and cytoplasm. The scale bar is 20 microns. **(B)** A co‐immunoprecipitation experiment was perfomed using cells stably expressing S1R-Apex. Anti-HA (control) and V5 antibody was used in the immunoprecipitiation. The co-precipitating proteins were analyzed by western blotting using antibodies against Bip and Calnexin.

SUPPLEMENTAL FIGURE S2
**(A–C)** Cells stably expressing S1R-Apex, either untreated or treated with Haloperidol or (+)-PTZ were fixed and processed for immunofluorescence using a V5 antibody. The signal is shown using a red to white LUT. **(D–F)** The same cells and treatment conditions were used. The cells were fixed and processed for immunofluorescence using antibodies against V5 (magenta) and LDLR (cyan). A merged image is also shown. The scale bar for images in panels A-F is 20 microns. **(G)** Co-localization between S1R (V5) and LDLR was analyzed using Imaris 10. A Pearson’s coefficient is shown. S1R partially co-localizes with LDLR. The level of co-localization is unchanged under conditions of ligand treatment. *n* = 12 images per condition. A one-way Anova was used for these analyses; ns = not significant.

SUPPLEMENTAL TABLE S1 List of S1R-Apex proximal proteins. Sheet 2 indicates S1R proximal proteins that have a “cell surface” GO annotation.

SUPPLEMENTAL TABLE S2 List of proteins that change their proximal association with S1R-Apex upon treatment with Haloperidol.

SUPPLEMENTAL TABLE S3 List of proteins that change their proximal association with S1R-Apex upon treatment with (+)-PTZ.

SUPPLEMENTAL TABLE S4 List of proteins that were detected in HeLa cell conditioned media.

SUPPLEMENTAL TABLE S5 List of proteins whose secretion is significantly altered upon treatment of cells with Haloperidol.

SUPPLEMENTAL TABLE S6 List of proteins whose secretion is significantly altered upon treatment of cells with (+)-PTZ.

SUPPLEMENTAL TABLE S7 List of S1R proximal proteins found in conditioned media.

## References

[B1] Abifadel M. Guerin M. Benjannet S. Rabes J. P. Le Goff W. Julia Z. (2012). Identification and characterization of new gain-of-function mutations in the PCSK9 gene responsible for autosomal dominant hypercholesterolemia. Atherosclerosis 223, 394–400. 10.1016/j.atherosclerosis.2012.04.006 22683120

[B2] Abifadel M. Rabes J. P. Devillers M. Munnich A. Erlich D. Junien C. (2009a). Mutations and polymorphisms in the proprotein convertase subtilisin kexin 9 (PCSK9) gene in cholesterol metabolism and disease. Hum. Mutat. 30, 520–529. 10.1002/humu.20882 19191301

[B3] Abifadel M. Rabes J. P. Jambart S. Halaby G. Gannage-Yared M. H. Sarkis A. (2009b). The molecular basis of familial hypercholesterolemia in Lebanon: Spectrum of LDLR mutations and role of PCSK9 as a modifier gene. Hum. Mutat. 30, E682–E691. 10.1002/humu.21002 19319977

[B4] Abifadel M. Varret M. Rabes J. P. Allard D. Ouguerram K. Devillers M. (2003). Mutations in PCSK9 cause autosomal dominant hypercholesterolemia. Nat. Genet. 34, 154–156. 10.1038/ng1161 12730697

[B5] Appenzeller-Herzog C. Hauri H. P. (2006). The ER-golgi intermediate compartment (ERGIC): In search of its identity and function. J. Cell Sci. 119, 2173–2183. 10.1242/jcs.03019 16723730

[B6] Arnold C. (2021). 11 clinical trials that will shape medicine in 2022. Nat. Med. 27, 2062–2064. 10.1038/s41591-021-01601-5 34893772

[B7] Bai T. Wang S. Zhao Y. Zhu R. Wang W. Sun Y. (2017). Haloperidol, a sigma receptor 1 antagonist, promotes ferroptosis in hepatocellular carcinoma cells. Biochem. biophysical Res. Commun. 491, 919–925. 10.1016/j.bbrc.2017.07.136 28756230

[B8] Balasuriya D. Stewart A. P. Edwardson J. M. (2013). The sigma-1 receptor interacts directly with GluN1 but not GluN2A in the GluN1/GluN2A NMDA receptor. J. Neurosci. 33, 18219–18224. 10.1523/JNEUROSCI.3360-13.2013 24227730 PMC3828470

[B9] Brown M. S. Goldstein J. L. (1986). A receptor-mediated pathway for cholesterol homeostasis. Science 232, 34–47. 10.1126/science.3513311 3513311

[B10] Brown M. S. Goldstein J. L. (1997). The SREBP pathway: Regulation of cholesterol metabolism by proteolysis of a membrane-bound transcription factor. Cell 89, 331–340. 10.1016/s0092-8674(00)80213-5 9150132

[B11] Cho K. F. Branon T. C. Udeshi N. D. Myers S. A. Carr S. A. Ting A. Y. (2020). Proximity labeling in mammalian cells with TurboID and split-TurboID. Nat. Protoc. 15, 3971–3999. 10.1038/s41596-020-0399-0 33139955

[B12] Cohen J. C. Boerwinkle E. Mosley T. H. Hobbs H. H. (2006). Sequence variations in PCSK9, low LDL, and protection against coronary heart disease. N. Engl. J. Med. 354, 1264–1272. 10.1056/NEJMoa054013 16554528

[B13] Das D. Persaud L. Dejoie J. Happy M. Brannigan O. De Jesus D. (2016). Tumor necrosis factor-related apoptosis-inducing ligand (TRAIL) activates caspases in human prostate cancer cells through sigma 1 receptor. Biochem. Biophys. Res. Commun. 470, 319–323. 10.1016/j.bbrc.2016.01.055 26792723

[B14] de Costa B. R. Bowen W. D. Hellewell S. B. Walker J. M. Thurkauf A. Jacobson A. E. (1989). Synthesis and evaluation of optically pure [3H]-(+)-pentazocine, a highly potent and selective radioligand for sigma receptors. FEBS Lett. 251, 53–58. 10.1016/0014-5793(89)81427-9 2568952

[B15] Fu Y. L. Zhang B. Mu T. W. (2019). LMAN1 (ERGIC-53) promotes trafficking of neuroreceptors. Biochem. Biophys. Res. Commun. 511, 356–362. 10.1016/j.bbrc.2019.02.053 30791981 PMC6431259

[B16] Gamayun I. O'Keefe S. Pick T. Klein M. C. Nguyen D. McKibbin C. (2019). Eeyarestatin compounds selectively enhance sec61-mediated Ca(2+) leakage from the endoplasmic reticulum. Cell Chem. Biol. 26, 571–583. 10.1016/j.chembiol.2019.01.010 30799222 PMC6483976

[B17] Geva M. Gershoni-Emek N. Naia L. Ly P. Mota S. Rego A. C. (2021). Neuroprotection of retinal ganglion cells by the sigma-1 receptor agonist pridopidine in models of experimental glaucoma. Sci. Rep. 11, 21975. 10.1038/s41598-021-01077-w 34753986 PMC8578336

[B18] Goldstein J. L. Brown M. S. (1977). The low-density lipoprotein pathway and its relation to atherosclerosis. Annu. Rev. Biochem. 46, 897–930. 10.1146/annurev.bi.46.070177.004341 197883

[B19] Gordon D. E. Jang G. M. Bouhaddou M. Xu J. Obernier K. White K. M.(2020). A SARS-CoV-2 protein interaction map reveals targets for drug repurposing. Nature 583, 459–468. 10.1038/s41586-020-2286-9 32353859 PMC7431030

[B20] Happy M. Dejoie J. Zajac C. K. Cortez B. Chakraborty K. Aderemi J. (2015). Sigma 1 Receptor antagonist potentiates the anti-cancer effect of p53 by regulating ER stress, ROS production, Bax levels, and caspase-3 activation. Biochem. Biophys. Res. Commun. 456, 683–688. 10.1016/j.bbrc.2014.12.029 25511708

[B21] Hauri H. P. Kappeler F. Andersson H. Appenzeller C. (2000). ERGIC-53 and traffic in the secretory pathway. J. Cell Sci. 113 (4), 587–596. 10.1242/jcs.113.4.587 10652252

[B22] Hayashi T. Su T. P. (2010). Cholesterol at the endoplasmic reticulum: Roles of the sigma-1 receptor chaperone and implications thereof in human diseases. Sub-cellular Biochem. 51, 381–398. 10.1007/978-90-481-8622-8_13 PMC3155710 20213551

[B23] Hayashi T. Su T. P. (2003). Intracellular dynamics of sigma-1 receptors (sigma(1) binding sites) in NG108-15 cells. J. Pharmacol. Exp. Ther. 306, 726–733. 10.1124/jpet.103.051292 12730356

[B24] Hayashi T. Su T. P. (2007). Sigma-1 receptor chaperones at the ER-mitochondrion interface regulate Ca(2+) signaling and cell survival. Cell 131, 596–610. 10.1016/j.cell.2007.08.036 17981125

[B25] Hayashi T. Su T. P. (2004). Sigma-1 receptor ligands: Potential in the treatment of neuropsychiatric disorders. CNS Drugs 18, 269–284. 10.2165/00023210-200418050-00001 15089113

[B26] Hayashi T. Su T. (2005). The sigma receptor: Evolution of the concept in neuropsychopharmacology. Curr. Neuropharmacol. 3, 267–280. 10.2174/157015905774322516 18369400 PMC2268997

[B27] Hicks L. Liu G. Ukken F. P. Lu S. Bollinger K. E. O'Connor-Giles K. (2015). Depletion or over-expression of Sh3px1 results in dramatic changes in cell morphology. Biol. Open 4, 1448–1461. 10.1242/bio.013755 26459243 PMC4728355

[B28] Hung V. Udeshi N. D. Lam S. S. Loh K. H. Cox K. J. Pedram K. (2016). Spatially resolved proteomic mapping in living cells with the engineered peroxidase APEX2. Nat. Protoc. 11, 456–475. 10.1038/nprot.2016.018 26866790 PMC4863649

[B29] Jiang G. Mysona B. Dun Y. Gnana-Prakasam J. P. Pabla N. Li W. (2006). Expression, subcellular localization, and regulation of sigma receptor in retinal muller cells. Investigative Ophthalmol. Vis. Sci. 47, 5576–5582. 10.1167/iovs.06-0608 PMC3724475 17122151

[B30] Lam S. S. Martell J. D. Kamer K. J. Deerinck T. J. Ellisman M. H. Mootha V. K. (2015). Directed evolution of APEX2 for electron microscopy and proximity labeling. Nat. Methods 12, 51–54. 10.1038/nmeth.3179 25419960 PMC4296904

[B31] Lang S. Pfeffer S. Lee P. H. Cavalie A. Helms V. Forster F. (2017). An update on Sec61 channel functions, mechanisms, and related diseases. Front. Physiol. 8, 887. 10.3389/fphys.2017.00887 29163222 PMC5672155

[B32] Lee S. Y. Kang M. G. Park J. S. Lee G. Ting A. Y. Rhee H. W. (2016). APEX fingerprinting reveals the subcellular localization of proteins of interest. Cell Rep. 15, 1837–1847. 10.1016/j.celrep.2016.04.064 27184847

[B33] Li L. He S. Liu Y. Yorio T. Ellis D. Z. (2021). Sigma-1R protects retinal ganglion cells in optic nerve crush model for glaucoma. Invest. Ophthalmol. Vis. Sci. 62, 17. 10.1167/iovs.62.10.17 PMC8375012 34406331

[B34] Linxweiler M. Schick B. Zimmermann R. (2017). Let's talk about Secs: Sec61, Sec62 and Sec63 in signal transduction, oncology and personalized medicine. Signal Transduct. Target Ther. 2, 17002. 10.1038/sigtrans.2017.2 29263911 PMC5661625

[B35] Lopez O. V. Gorantla S. Segarra A. C. Andino Norat M. C. Alvarez M. Skolasky R. L. (2019). Sigma-1 receptor antagonist (BD1047) decreases cathepsin B secretion in HIV-infected macrophages exposed to cocaine. J. Neuroimmune Pharmacol. 14, 226–240. 10.1007/s11481-018-9807-4 30306495 PMC6488453

[B36] Lorbek G. Lewinska M. Rozman D. (2012). Cytochrome P450s in the synthesis of cholesterol and bile acids--from mouse models to human diseases. FEBS J. 279, 1516–1533. 10.1111/j.1742-4658.2011.08432.x 22111624

[B37] Maher C. M. Thomas J. D. Haas D. A. Longen C. G. Oyer H. M. Tong J. Y. (2018). Small-molecule Sigma1 modulator induces autophagic degradation of PD-L1. Mol. Cancer Res. 16, 243–255. 10.1158/1541-7786.MCR-17-0166 29117944

[B38] Maurice T. (2021). Bi-phasic dose response in the preclinical and clinical developments of sigma-1 receptor ligands for the treatment of neurodegenerative disorders. Expert Opin. Drug Discov. 16, 373–389. 10.1080/17460441.2021.1838483 33070647

[B39] Maurice T. Su T. P. (2009). The pharmacology of sigma-1 receptors. Pharmacol. Ther. 124, 195–206. 10.1016/j.pharmthera.2009.07.001 19619582 PMC2785038

[B40] Mavlyutov T. A. Epstein M. Guo L. W. (2015). Subcellular localization of the sigma-1 receptor in retinal neurons - an electron microscopy study. Sci. Rep. 5, 10689. 10.1038/srep10689 26033680 PMC4649997

[B41] Mavlyutov T. A. Guo L. W. Epstein M. L. Ruoho A. E. (2015). Role of the sigma-1 receptor in amyotrophic lateral Sclerosis (ALS). J. Pharmacol. Sci. 127, 10–16. 10.1016/j.jphs.2014.12.013 25704013 PMC4489701

[B42] Mavlyutov T. A. Yang H. Epstein M. L. Ruoho A. E. Yang J. Guo L. W. (2017). APEX2-enhanced electron microscopy distinguishes sigma-1 receptor localization in the nucleoplasmic reticulum. Oncotarget 8, 51317–51330. 10.18632/oncotarget.17906 28881650 PMC5584251

[B43] McGilvray P. T. Anghel S. A. Sundaram A. Zhong F. Trnka M. J. Fuller J. R. (2020). An ER translocon for multi-pass membrane protein biogenesis. Elife 9, e56889. 10.7554/eLife.56889 32820719 PMC7505659

[B44] Mei J. Pasternak G. W. (2007). Modulation of brainstem opiate analgesia in the rat by sigma 1 receptors: A microinjection study. J. Pharmacol. Exp. Ther. 322, 1278–1285. 10.1124/jpet.107.121137 17545312

[B45] Mysona B. A. Zhao J. Smith S. Bollinger K. E. (2018). Relationship between Sigma-1 receptor and BDNF in the visual system. Exp. Eye Res. 167, 25–30. 10.1016/j.exer.2017.10.012 29031856 PMC5757370

[B46] Navarro G. Moreno E. Aymerich M. Marcellino D. McCormick P. J. Mallol J. (2010). Direct involvement of sigma-1 receptors in the dopamine D1 receptor-mediated effects of cocaine. Proc. Natl. Acad. Sci. U. S. A. 107, 18676–18681. 10.1073/pnas.1008911107 20956312 PMC2972946

[B47] Nguyen L. Lucke-Wold B. P. Mookerjee S. A. Cavendish J. Z. Robson M. J. Scandinaro A. L. (2015). Role of sigma-1 receptors in neurodegenerative diseases. J. Pharmacol. Sci. 127, 17–29. 10.1016/j.jphs.2014.12.005 25704014

[B48] Oceguera-Yanez F. Kim S. I. Matsumoto T. Tan G. W. Xiang L. Hatani T. (2016). Engineering the AAVS1 locus for consistent and scalable transgene expression in human iPSCs and their differentiated derivatives. Methods 101, 43–55. 10.1016/j.ymeth.2015.12.012 26707206

[B49] Ola M. S. Moore P. Maddox D. El-Sherbeny A. Huang W. Roon P. (2002). Analysis of sigma receptor (sigmaR1) expression in retinal ganglion cells cultured under hyperglycemic conditions and in diabetic mice. Brain Res. Mol. Brain Res. 107, 97–107. 10.1016/s0169-328x(02)00444-8 12425939 PMC3773709

[B50] Palmer C. P. Mahen R. Schnell E. Djamgoz M. B. Aydar E. (2007). Sigma-1 receptors bind cholesterol and remodel lipid rafts in breast cancer cell lines. Cancer Res. 67, 11166–11175. 10.1158/0008-5472.CAN-07-1771 18056441

[B51] Reis G. Dos Santos Moreira-Silva E. A. Silva D. C. M. Thabane L. Milagres A. C. Ferreira T. S. (2022). Effect of early treatment with fluvoxamine on risk of emergency care and hospitalisation among patients with COVID-19: The TOGETHER randomised, platform clinical trial. Lancet Glob. Health 10, e42–e51. 10.1016/S2214-109X(21)00448-4 34717820 PMC8550952

[B52] Rhee H. W. Zou P. Udeshi N. D. Martell J. D. Mootha V. K. Carr S. A. (2013). Proteomic mapping of mitochondria in living cells via spatially restricted enzymatic tagging. Science 339, 1328–1331. 10.1126/science.1230593 23371551 PMC3916822

[B53] Sabino V. Cottone P. Parylak S. L. Steardo L. Zorrilla E. P. (2009). Sigma-1 receptor knockout mice display a depressive-like phenotype. Behav. Brain Res. 198, 472–476. 10.1016/j.bbr.2008.11.036 19100292 PMC2667953

[B54] Samavarchi-Tehrani P. Samson R. Gingras A. C. (2020). Proximity dependent biotinylation: Key enzymes and adaptation to proteomics approaches. Mol. Cell Proteomics 19, 757–773. 10.1074/mcp.R120.001941 32127388 PMC7196579

[B55] Sambo D. O. Lin M. Owens A. Lebowitz J. J. Richardson B. Jagnarine D. A. (2017). The sigma-1 receptor modulates methamphetamine dysregulation of dopamine neurotransmission. Nat. Commun. 8, 2228. 10.1038/s41467-017-02087-x 29263318 PMC5738444

[B56] Schindler R. Itin C. Zerial M. Lottspeich F. Hauri H. P. (1993). ERGIC-53, a membrane protein of the ER-Golgi intermediate compartment, carries an ER retention motif. Eur. J. Cell Biol. 61, 1–9. 8223692

[B57] Schmidt H. R. Betz R. M. Dror R. O. Kruse A. C. (2018). Structural basis for σ1 receptor ligand recognition. Nat. Struct. Mol. Biol. 25, 981–987. 10.1038/s41594-018-0137-2 30291362 PMC6261271

[B58] Schmidt H. R. Kruse A. C. (2019). The molecular function of sigma receptors: Past, present, and future. Trends Pharmacol. Sci. 40, 636–654. 10.1016/j.tips.2019.07.006 31387763 PMC6748033

[B59] Schmidt H. R. Zheng S. Gurpinar E. Koehl A. Manglik A. Kruse A. C. (2016). Crystal structure of the human σ1 receptor. Nature 532, 527–530. 10.1038/nature17391 27042935 PMC5550834

[B60] Seyfried N. T. Dammer E. B. Swarup V. Nandakumar D. Duong D. M. Yin L. (2017). A multi-network approach identifies protein-specific Co-expression in asymptomatic and symptomatic alzheimer's disease. Cell Syst. 4, 60–72. 10.1016/j.cels.2016.11.006 27989508 PMC5269514

[B61] Shader R. (1994). “Approaches to the treatment of schizophrenia,” in Manual of psychiatric therapeutics. Editor Shader R. I. (Boston: Little Brown and Co) 311–336.

[B62] Shechter I. Klinger E. Rucker M. L. Engstrom R. G. Spirito J. A. Islam M. A. (1992). Solubilization, purification, and characterization of a truncated form of rat hepatic squalene synthetase. J. Biol. Chem. 267, 8628–8635. 10.1016/s0021-9258(18)42489-1 1569107

[B63] Soucek S. Zeng Y. Bellur D. L. Bergkessel M. Morris K. J. Deng Q. (2016). The evolutionarily-conserved polyadenosine RNA binding protein, Nab2, cooperates with splicing machinery to regulate the fate of pre-mRNA. Mol. Cell. Biol. 36, 2697–2714. 10.1128/MCB.00402-16 27528618 PMC5064217

[B64] Su T. P. Hayashi T. Maurice T. Buch S. Ruoho A. E. (2010). The sigma-1 receptor chaperone as an inter-organelle signaling modulator. Trends Pharmacol. Sci. 31, 557–566. 10.1016/j.tips.2010.08.007 20869780 PMC2993063

[B65] Su T. P. Su T. C. Nakamura Y. Tsai S. Y. (2016). The sigma-1 receptor as a pluripotent modulator in living systems. Trends Pharmacol. Sci. 37, 262–278. 10.1016/j.tips.2016.01.003 26869505 PMC4811735

[B66] Trinkle-Mulcahy L. (2019). Recent advances in proximity-based labeling methods for interactome mapping. F1000Res 8, F1000. 10.12688/f1000research.16903.1 PMC6357996 30774936

[B67] Tsai S. Y. Chuang J. Y. Tsai M. S. Wang X. F. Xi Z. X. Hung J. J. (2015). Sigma-1 receptor mediates cocaine-induced transcriptional regulation by recruiting chromatin-remodeling factors at the nuclear envelope. Proc. Natl. Acad. Sci. U. S. A. 112, E6562–E6570. 10.1073/pnas.1518894112 26554014 PMC4664336

[B68] Vavers E. Zvejniece L. Maurice T. Dambrova M. (2019). Allosteric modulators of sigma-1 receptor: A review. Front. Pharmacol. 10, 223. 10.3389/fphar.2019.00223 30941035 PMC6433746

[B69] Vela J. M. (2020). Repurposing sigma-1 receptor ligands for COVID-19 therapy? Front. Pharmacol. 11, 582310. 10.3389/fphar.2020.582310 33364957 PMC7751758

[B70] Walker J. M. Bowen W. D. Walker F. O. Matsumoto R. R. De Costa B. Rice K. C. (1990). Sigma receptors: Biology and function. Pharmacol. Rev. 42, 355–402. 1964225

[B71] Wang J. Saul A. Roon P. Smith S. B. (2016). Activation of the molecular chaperone, sigma 1 receptor, preserves cone function in a murine model of inherited retinal degeneration. Proc. Natl. Acad. Sci. U. S. A. 113, E3764–E3772. 10.1073/pnas.1521749113 27298364 PMC4932934

[B72] Wang Q. Li L. Ye Y. (2008). Inhibition of p97-dependent protein degradation by Eeyarestatin I. J. Biol. Chem. 283, 7445–7454. 10.1074/jbc.M708347200 18199748 PMC2276333

[B73] Wang Q. Shinkre B. A. Lee J. G. Weniger M. A. Liu Y. Chen W. (2010). The ERAD inhibitor Eeyarestatin I is a bifunctional compound with a membrane-binding domain and a p97/VCP inhibitory group. PLoS One 5, e15479. 10.1371/journal.pone.0015479 21124757 PMC2993181

[B74] Ward T. H. Polishchuk R. S. Caplan S. Hirschberg K. Lippincott-Schwartz J. (2001). Maintenance of Golgi structure and function depends on the integrity of ER export. J. Cell Biol. 155, 557–570. 10.1083/jcb.200107045 11706049 PMC2198855

[B75] Weinreich M. Frishman W. H. (2014). Antihyperlipidemic therapies targeting PCSK9. Cardiol. Rev. 22, 140–146. 10.1097/CRD.0000000000000014 24407047

[B76] Ye N. Qin W. Tian S. Xu Q. Wold E. A. Zhou J. (2020). Small molecules selectively targeting sigma-1 receptor for the treatment of neurological diseases. J. Med. Chem. 63, 15187–15217. 10.1021/acs.jmedchem.0c01192 33111525

[B77] Zhemkov V. Ditlev J. A. Lee W. R. Wilson M. Liou J. Rosen M. K. (2021b). The role of sigma 1 receptor in organization of endoplasmic reticulum signaling microdomains. Elife 10, e65192. 10.7554/eLife.65192 33973848 PMC8112866

[B78] Zhemkov V. Geva M. Hayden M. R. Bezprozvanny I. (2021a). Sigma-1 receptor (S1R) interaction with cholesterol: Mechanisms of S1R activation and its role in neurodegenerative diseases. Int. J. Mol. Sci. 22, 4082. 10.3390/ijms22084082 33920913 PMC8071319

