# Supplementary figures and images for: Defining the ligand-dependent proximatome of the sigma 1 receptor
Source: Front Cell Dev Biol. 2023 Jun 7;11:1045759. doi: 10.3389/fcell.2023.1045759 (PMC10284605; doi:10.3389/fcell.2023.1045759)

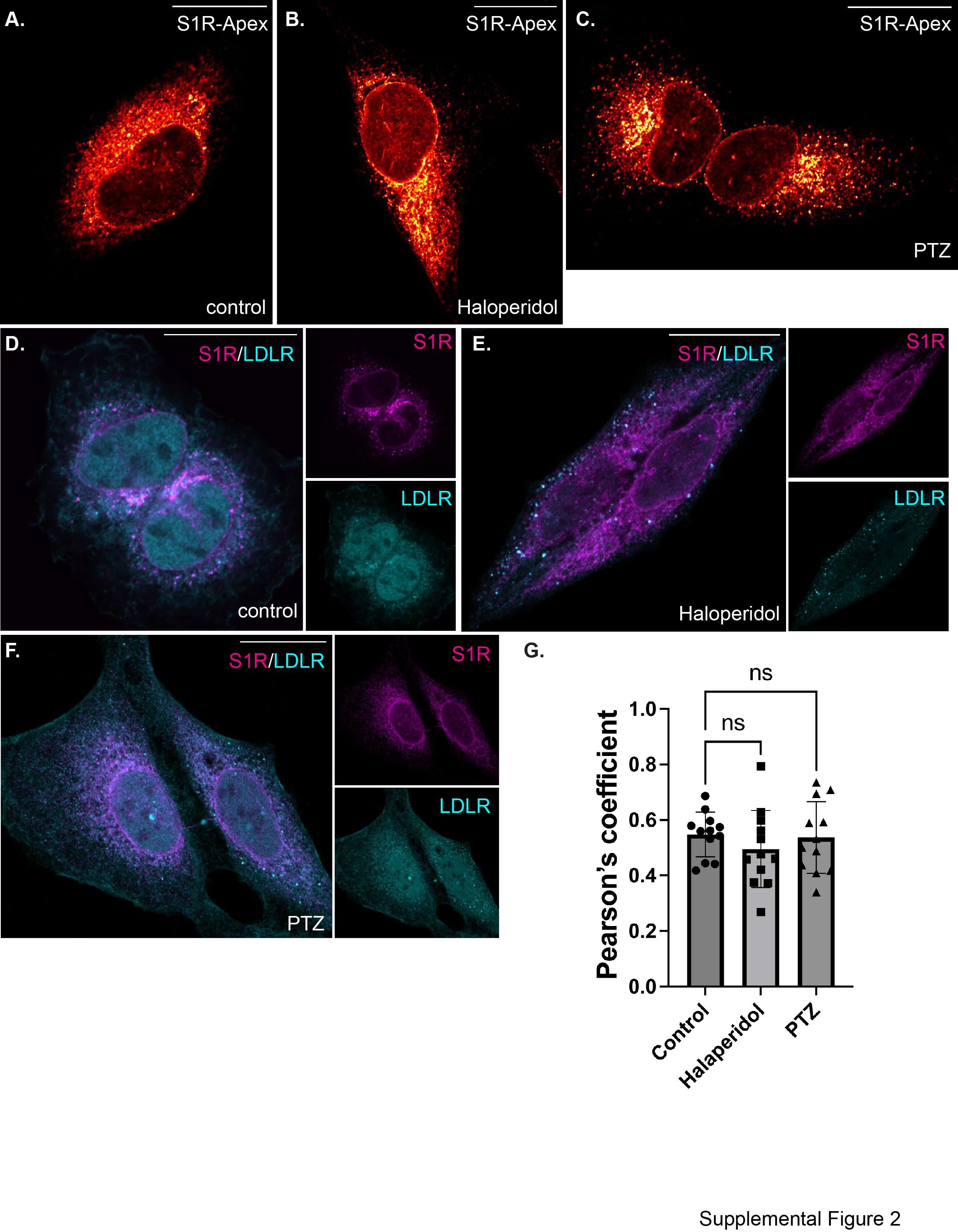

Supplement: Supplementary file 3 [file Image2.jpg]

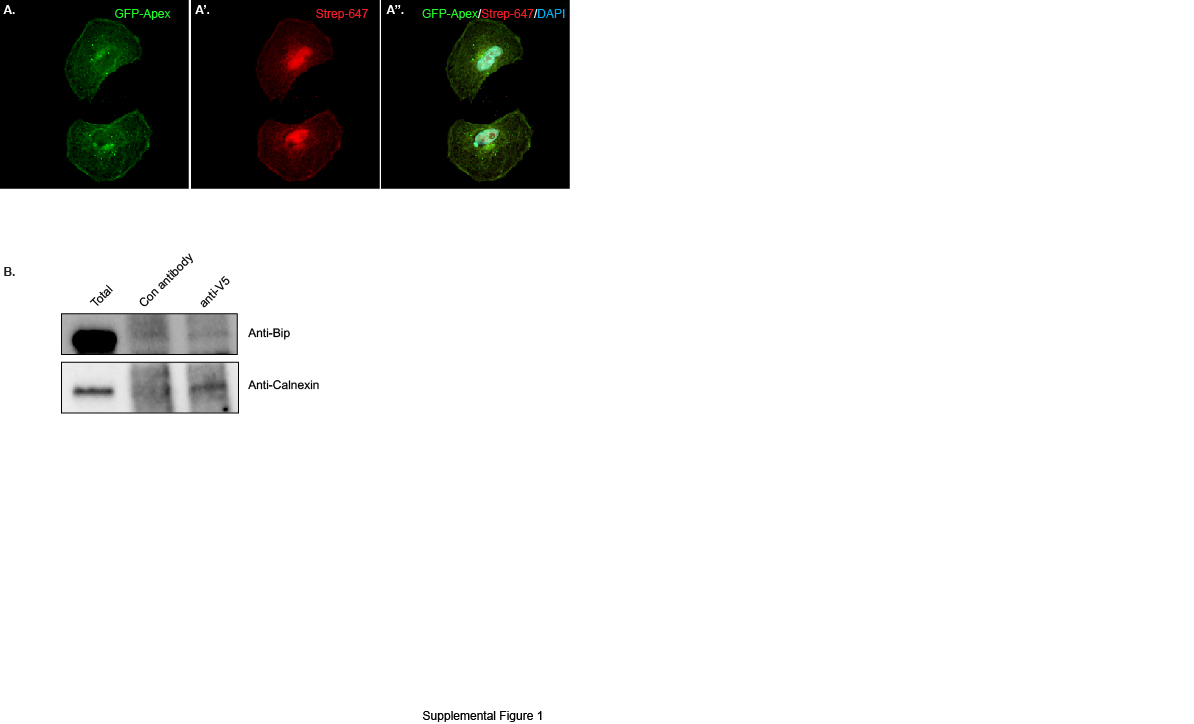

Supplement: Supplementary file 4 [file Image1.JPEG]
